# Supplementary material for: Hydroclimate shapes photosynthetic sensitivity to cloud cover across global terrestrial ecosystems
Source: Nat Commun. 2026 Feb 12;17:1646. doi: 10.1038/s41467-026-69480-3 (PMC12905301; doi:10.1038/s41467-026-69480-3)
Supplement: Supplementary file 1 — Supplementary Information [file 41467_2026_69480_MOESM1_ESM.pdf]

Supplementary Information for

Hydroclimate shapes photosynthetic sensitivity to cloud cover across global terrestrial ecosystems

Hao Luo[1*], Ana Bastos[2], Markus Reichstein[3], Gregory Duveiller[3], Jan Kretzschmar[1], Johannes Quaas[1,4]

[1]Leipzig Institute for Meteorology, Leipzig University, 04103, Leipzig, Germany

[2]Institute for Earth System Science and Remote Sensing, Leipzig University, 04103, Leipzig, Germany

[3]Max Planck Institute for Biogeochemistry, 07745, Jena, Germany

[4]German Centre for Integrative Biodiversity Research (iDiv) Halle-Jena-Leipzig, 04103, Leipzig, Germany

[*]Corresponding author. Email: hao.luo@uni-leipzig.de

This supplementary information includes:

Supplementary texts

Supplementary Figure 1 to Figure 37

Supplementary Table 1 to Table 3

Supplementary references

Sensitivity analysis

Examining different vegetation types

By categorizing land cover into nine different vegetation types (Methods, Supplementary Fig. 25), it is evident that variations in HI still influence the GPP^*-to-CF^* sensitivity for a certain ecosystem, except for deciduous broadleaf forests due to few samples and for evergreen broadleaf forests (Supplementary Fig. 28). At the same time, the decreasing GPP^*-to-CF^* sensitivity with HI across different ecosystems shows an anomaly in the evergreen broadleaf forests, where the average sensitivity is not the most negative despite the average HI being the highest (Supplementary Fig. 28j). This suggests that the impact of clouds on vegetation dynamics is not fully realized in evergreen broadleaf forests, where the canopy is the densest. In these forests, the direct component of cloud radiative effects can only effectively influence the uppermost layer of the dense canopy, while photosynthesis in such forests is more likely sensitive to diffuse radiation, which may increase with higher CF and can thereby enhance GPP.

Comparison with independent datasets

The same spatial patterns of photosynthetic sensitivity to CF are drawn from other datasets as well. In addition to matching CF data from ERA5 for each FLUXNET site, we also incorporate CF data from the nearest grid of the daily 1° × 1° MODIS/Terra observations. Although the available time span for some sites is shortened and the matched data have a coarser spatial resolution when using MODIS-observed CF data, the conclusions remain robust and consistent (Supplementary Fig. 8a and b). For the global estimates, to minimize potential confounding effects from correlations between meteorological factors and cloud properties, our main analysis relies on the FLUXCOM-RS GPP product, which is purely derived from FLUXNET upscaling and remote sensing observations without the input of meteorological variables. We additionally provide GPP^*-to-CF^* sensitivity estimates using GPP data from two alternative FLUXCOM products that incorporate meteorological input: FLUXCOM-RS_METEO_ERA5 and FLUXCOM-X-BASE. The sensitivities derived from these two additional FLUXCOM products are spatially consistent with those from FLUXCOM-RS, both exhibiting a similar functional dependence on HI (Supplementary Figs. 29 and 30). It should be noted, however, that the sensitivity estimated from FLUXCOM-X-BASE generally shows higher values than that from FLUXCOM-RS on a global scale. This difference may partly reflect the limited representation and associated uncertainties of capturing

water-related processes in the FLUXCOM framework. As documented by ref.[1], FLUXCOM-X-BASE systematically shows higher evapotranspiration (ET) than FLUXCOM-RS, and its agreement with sun-induced fluorescence (SIF) from the Sentinel-5P TROPOMI instrument is weaker when compared to RS.

Besides the global scale analysis based on the FLUXCOM framework, other proxies of photosynthesis are adopted to examine the response of vegetation dynamics to CF. These include GPP from MODIS/Terra, SIF from the global dataset of solar-induced chlorophyll fluorescence (GOSIF), and the vegetation optical depth (VOD) night-to-day ratio (VODndr) from the Version 3 global land parameter data record (LPDR v3) X-band VOD dataset (for more details, see "Methods" section). From different perspectives, these proxies consistently show that the sensitivity of photosynthesis to CF is spatially shaped by hydroclimate (Supplementary Fig. 31). Here, VOD is additionally incorporated because other optical remote sensing observations may be affected by clouds[2], even though 8-daily averages are used to minimize this impact. In contrast, VOD, derived from microwave retrievals, effectively mitigates the uncertainties caused by cloud obstruction[3]. Instead of using VOD data directly, we use VODndr to better represent photosynthesis, as CO_2 uptake in vegetation is regulated by stomatal activity, while VOD fluctuates between day and night due to the inconsistency in stomatal function (Methods). Overall, all global scale proxies of photosynthesis are systematically validated against FLUXNET site-level GPP observations at an 8-daily temporal resolution to ensure data reliability and robustness (Supplementary Fig. 24).

For the estimates of annual-scale GPP-to-CF sensitivity (equation (7), Methods), in addition to being based on GPP from FLUXCOM-RS, we also derive it based on GPP data from MODIS and FLUXCOM-X-BASE (Supplementary Figs. 32 and 33). Both estimations exhibit spatial consistency, with the proportion of pixels showing consistent signs exceeding 80% when compared to the analysis based on FLUXCOM-RS GPP. Based on these estimates of annual-scale GPP-to-CF sensitivity from MODIS and FLUXCOM-X-BASE GPP datasets, similar spatial shifts in CF-driven GPP are also derived (Supplementary Figs. 34–37). However, there is a difference in the spatial shifts observed when using GPP data from FLUXCOM-X-BASE, which shows a slower decline in CF-driven GPP over humid regions compared to arid regions (Supplementary Fig. 36). This is primarily due to the positive GPP-to-CF sensitivity observed in evergreen broadleaf forests for FLUXCOM-X-BASE data (Supplementary Fig. 33c). In other words, in evergreen broadleaf

forests, the weaker direct component of radiative effects seen in FLUXCOM-RS (Supplementary Fig. 28j) is more pronounced in FLUXCOM-X-BASE, leading to a positive GPP-to-CF sensitivity. This difference can partly be attributed to uncertainties in interannual variability. As documented by ref.[1], FLUXCOM-X-BASE exhibits weaker interannual variability in GPP compared to FLUXCOM-RS (0.575 vs. 1.023 Pg C yr^{-1}), which may contribute to the differences in annual-scale GPP-to-CF sensitivity.

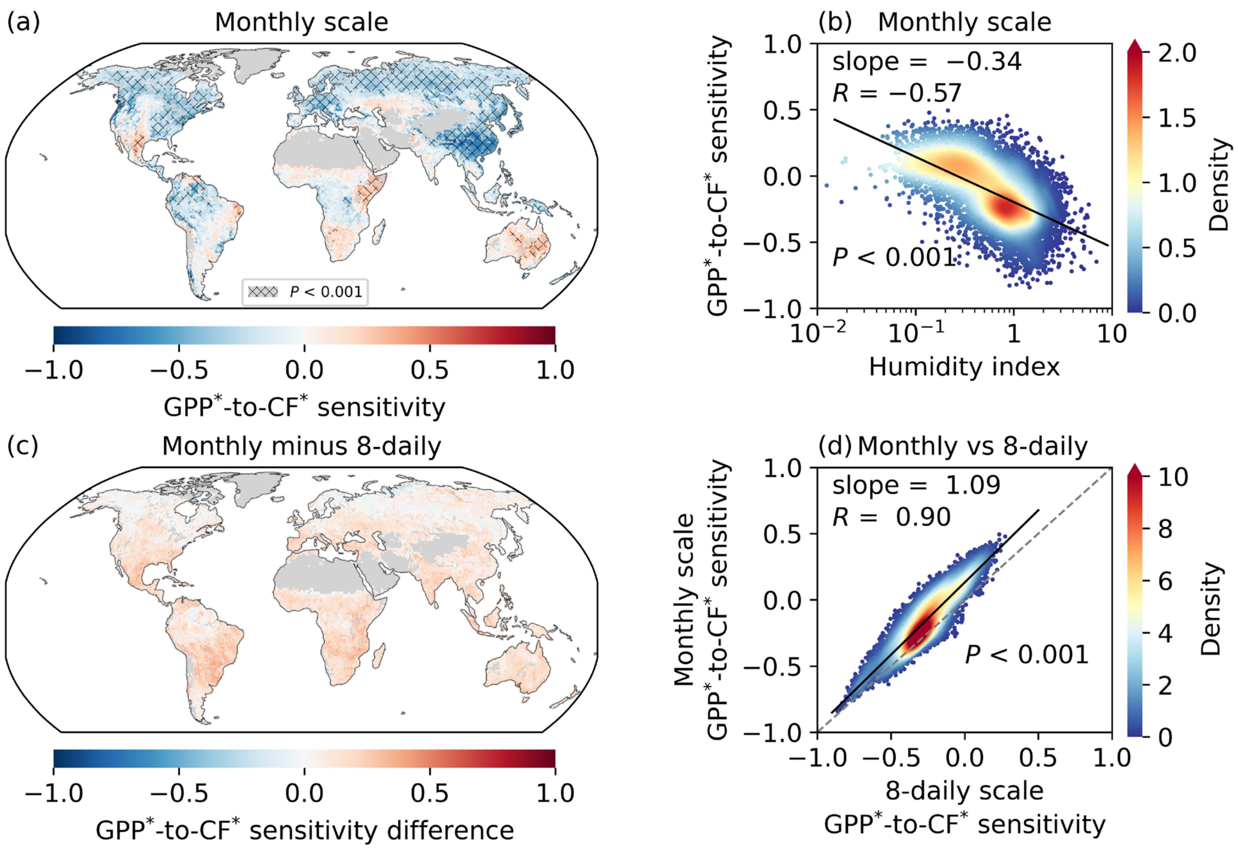

Supplementary Figure 1. Humidity index (HI) spatially shapes the sensitivity of gross primary productivity (GPP) to cloud fraction (CF) across global ecosystems on a monthly scale. (**a**) and (**b**) As in Fig. 1c and d but for monthly scale analysis. (**c**) The difference map between (**a**) and Fig. 1c. (**d**) Density plot showing the comparison between the monthly scale and 8-daily scale GPP^*-to-CF^* sensitivity across grids in (**c**). The linear regression is represented by the black line, with the texts displaying the slope of the linear fit, correlation coefficient (*R*), and *P*-value from a Student's t test. The colour bar shows the Gaussian kernel density estimate. The 1:1 line is marked with a grey dotted line.

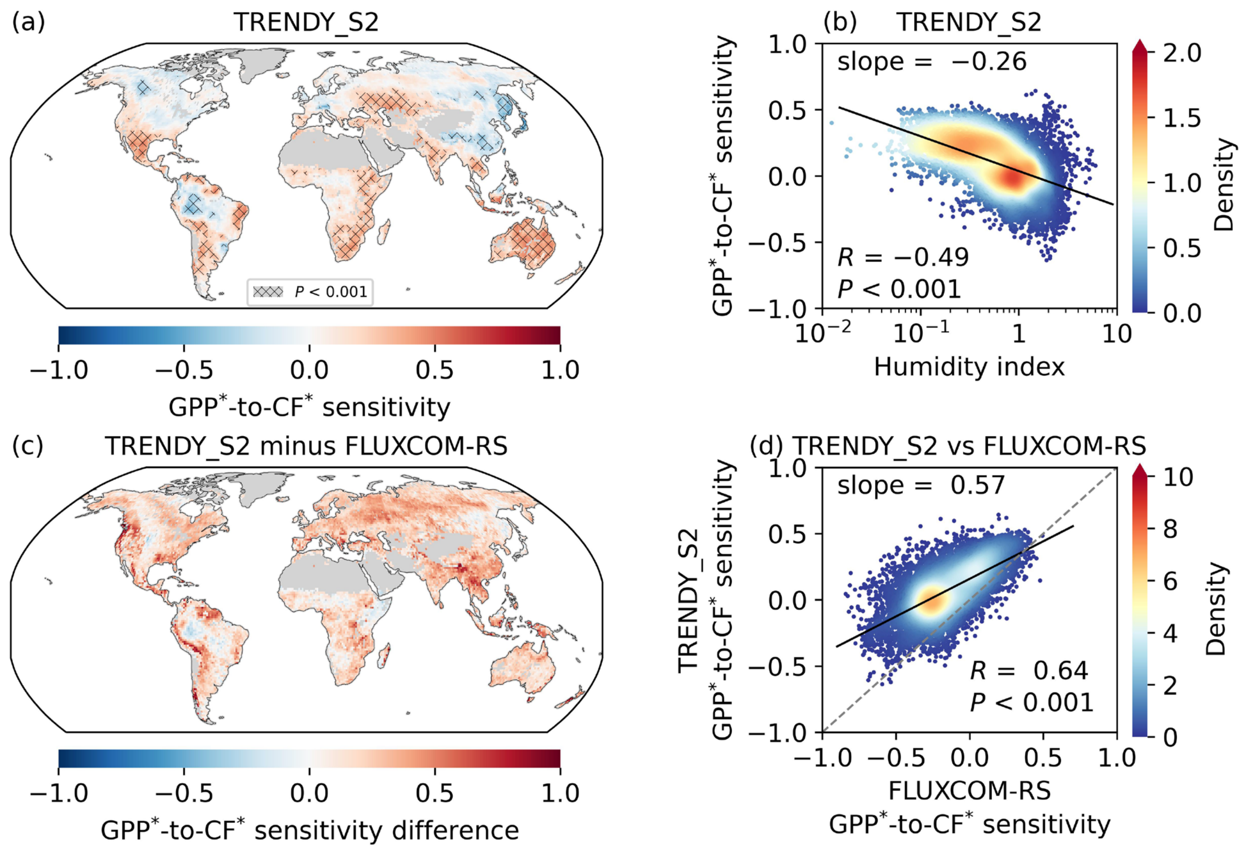

Supplementary Figure 2. Humidity index (HI) spatially shapes the sensitivity of gross primary productivity (GPP) to cloud fraction (CF) across global ecosystems on a monthly scale, derived from GPP simulated by TRENDYv12 and CF data from the Moderate Resolution Imaging Spectroradiometer (MODIS) onboard Terra. (**a**) and (**b**) As in Supplementary Fig. 1a and b but using ensemble mean GPP simulated from 20 dynamic global vegetation models. (**c**) The difference map between (**a**) and Supplementary Fig. 1a. (**d**) Density plot showing the comparison between the calculated GPP*-to-CF* sensitivity based on GPP data from TRENDYv12 and FLUXCOM-RS across grids in (**c**). The linear regression is represented by the black line, with the texts displaying the slope of the linear fit, correlation coefficient (R), and P-value from a Student's t test. The colour bar shows the Gaussian kernel density estimate. The 1:1 line is marked with a grey dotted line. Besides, the analysis is based on monthly scale data.

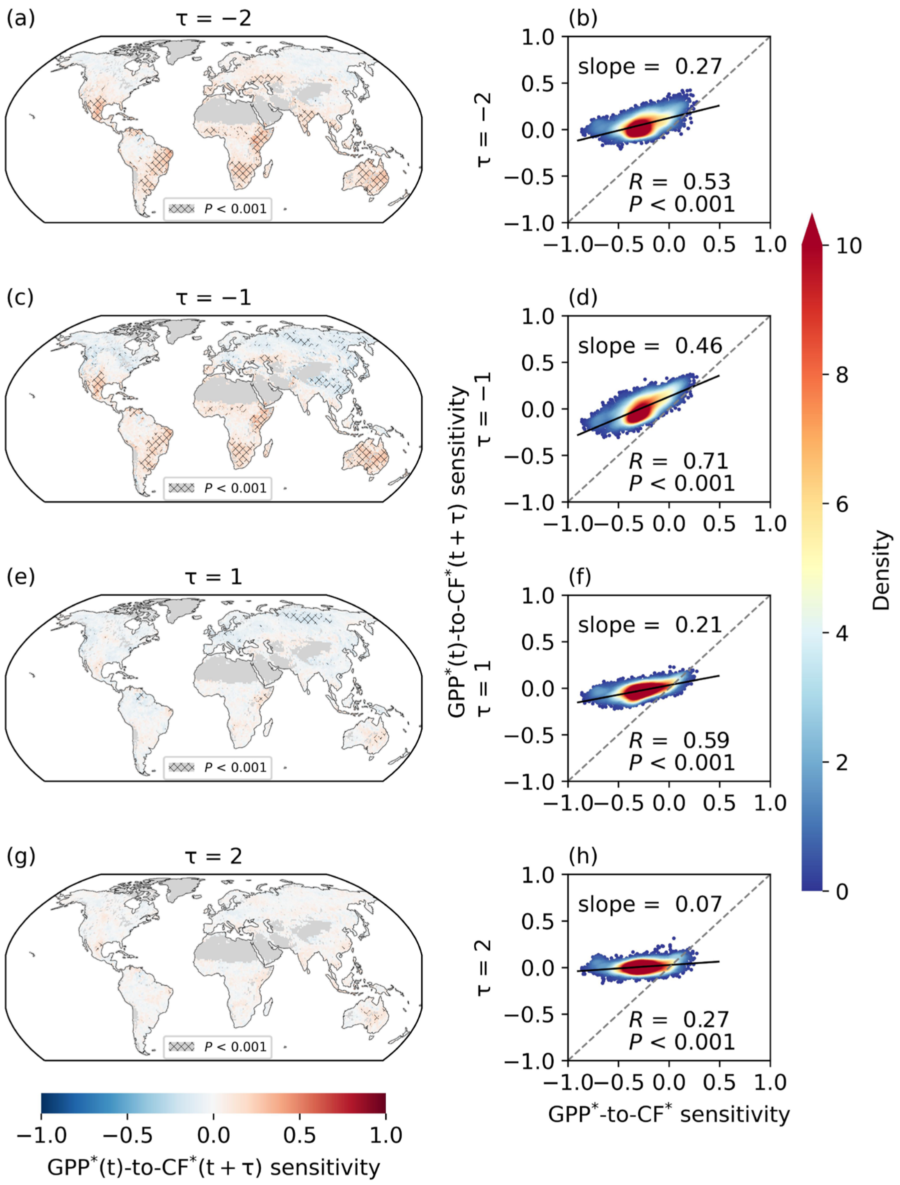

Supplementary Figure 3. Time-lagged sensitivities of gross primary productivity (GPP) to cloud fraction (CF). Map of time-lagged $GPP^*(t)$-to-$CF^*(t+\tau)$ sensitivity (equation (8), Methods) and comparisons against GPP^*-to-CF^* sensitivity ($\tau = 0$), with τ equalling (**a**) and (**b**) −2, (**c**) and (**d**) −1, (**e**) and (**f**) 1, and (**g**) and (**h**) 2, respectively. The cross-hatched areas represent regions where *P*-value from a Student's t-test is less than 0.001. All comparisons against GPP^*-to-CF^* sensitivity are presented as density plots across grids, with the colour bar showing the Gaussian kernel density estimate. The linear regressions are represented by the black lines, with the texts displaying the slope of the linear fit, correlation coefficient (*R*), and *P*-value from a Student's t test. The 1:1 line in each density subplot is marked with a grey dotted line.

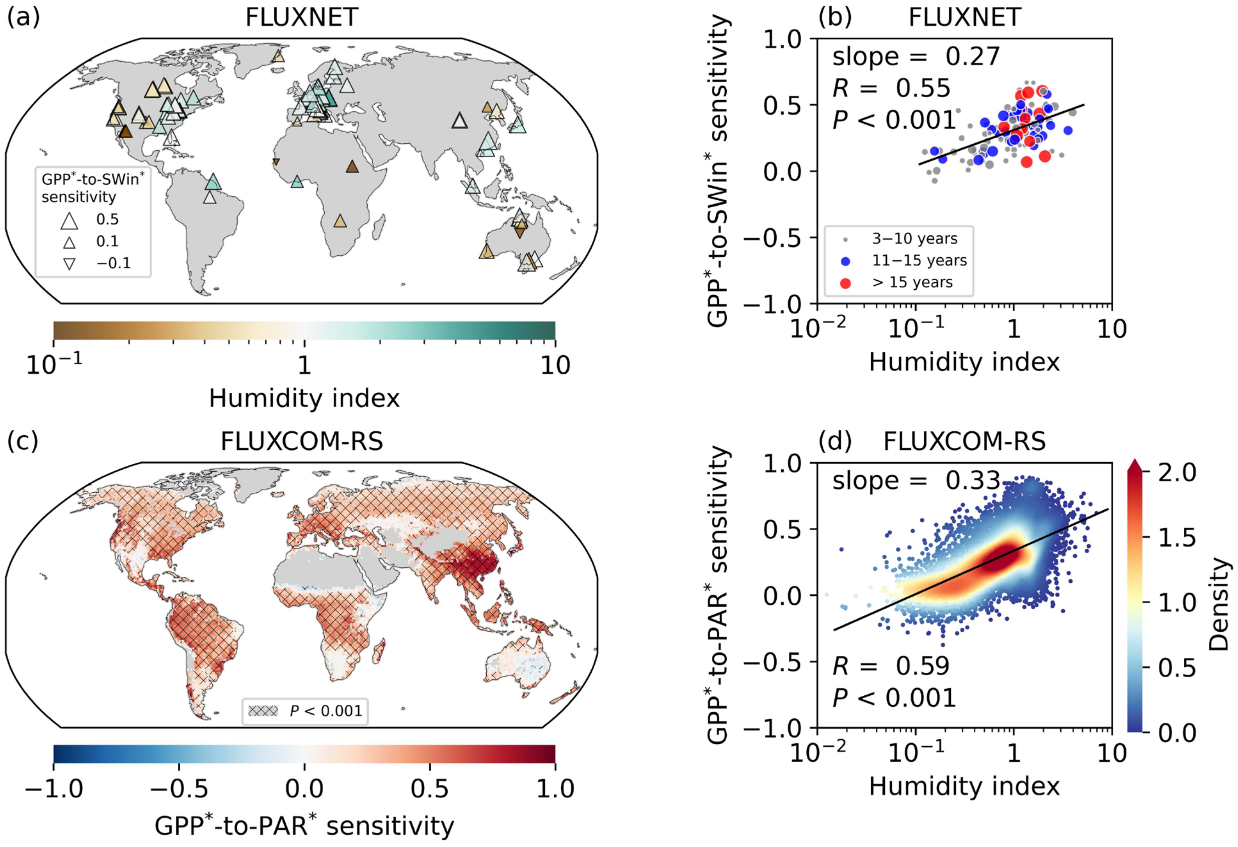

Supplementary Figure 4. Sensitivity of gross primary productivity (GPP) to photosynthetically active radiation (PAR) and its relationship with humidity index (HI). Same as Fig. 1 but for PAR rather than cloud fraction (CF). For the FLUXNET, surface incoming shortwave radiation (SWin) is used as a proxy for PAR.

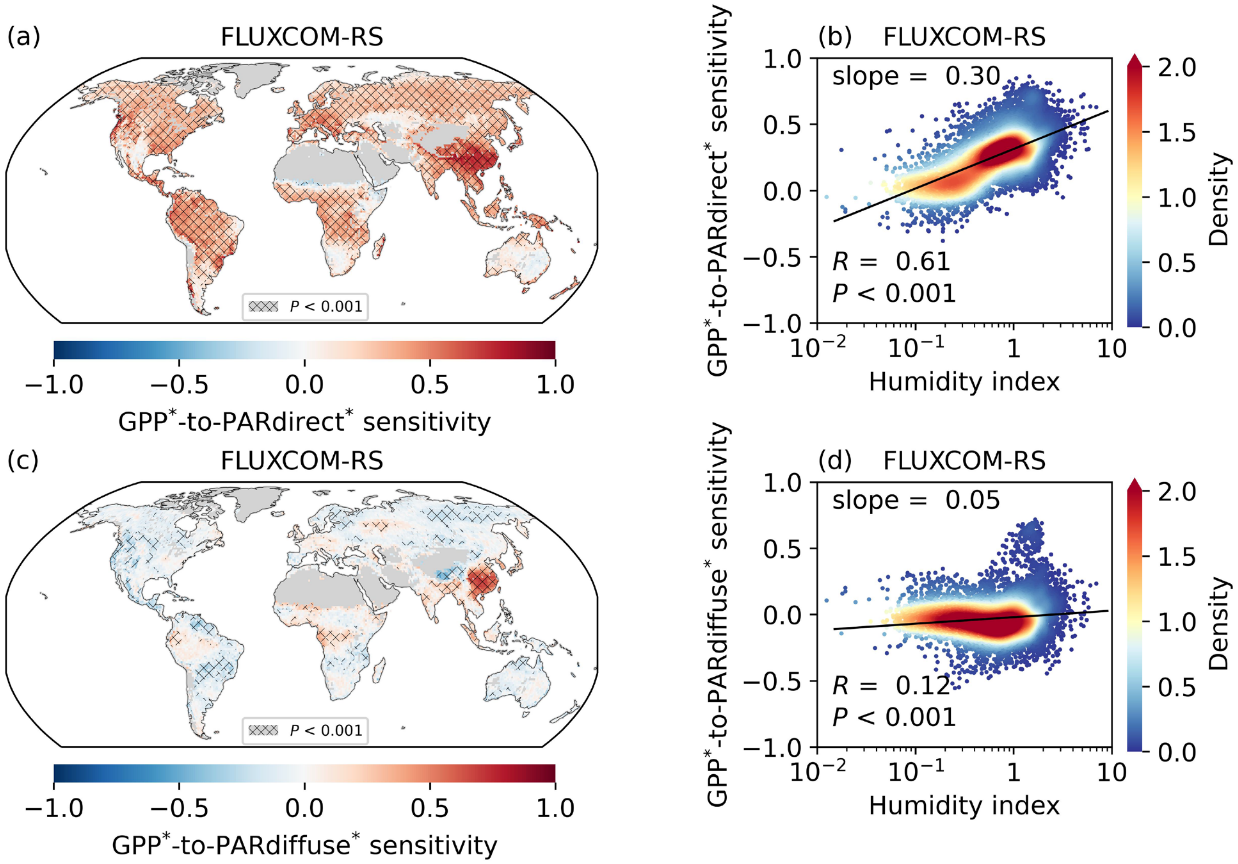

Supplementary Figure 5. Sensitivity of gross primary productivity (GPP) to direct and diffuse photosynthetically active radiation (PAR) and their relationships with humidity index (HI). (**a**) and (**b**) As in Supplementary Fig. 4a and b but for direct PAR. (**c**) and (**d**) As in Supplementary Fig. 4a and b but for diffuse PAR.

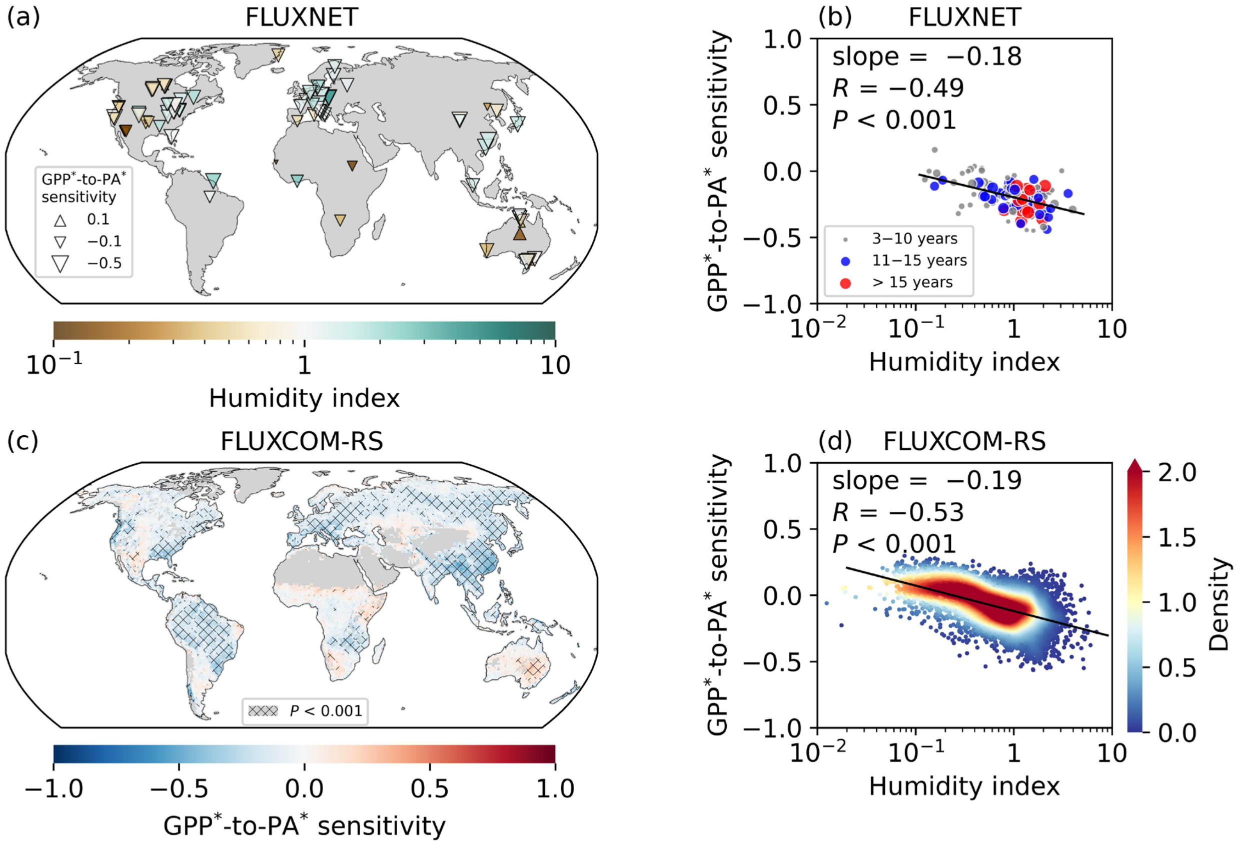

Supplementary Figure 6. Sensitivity of gross primary productivity (GPP) to precipitation amount (PA) and its relationship with humidity index (HI). Same as Fig. 1 but for PA rather than cloud fraction (CF).

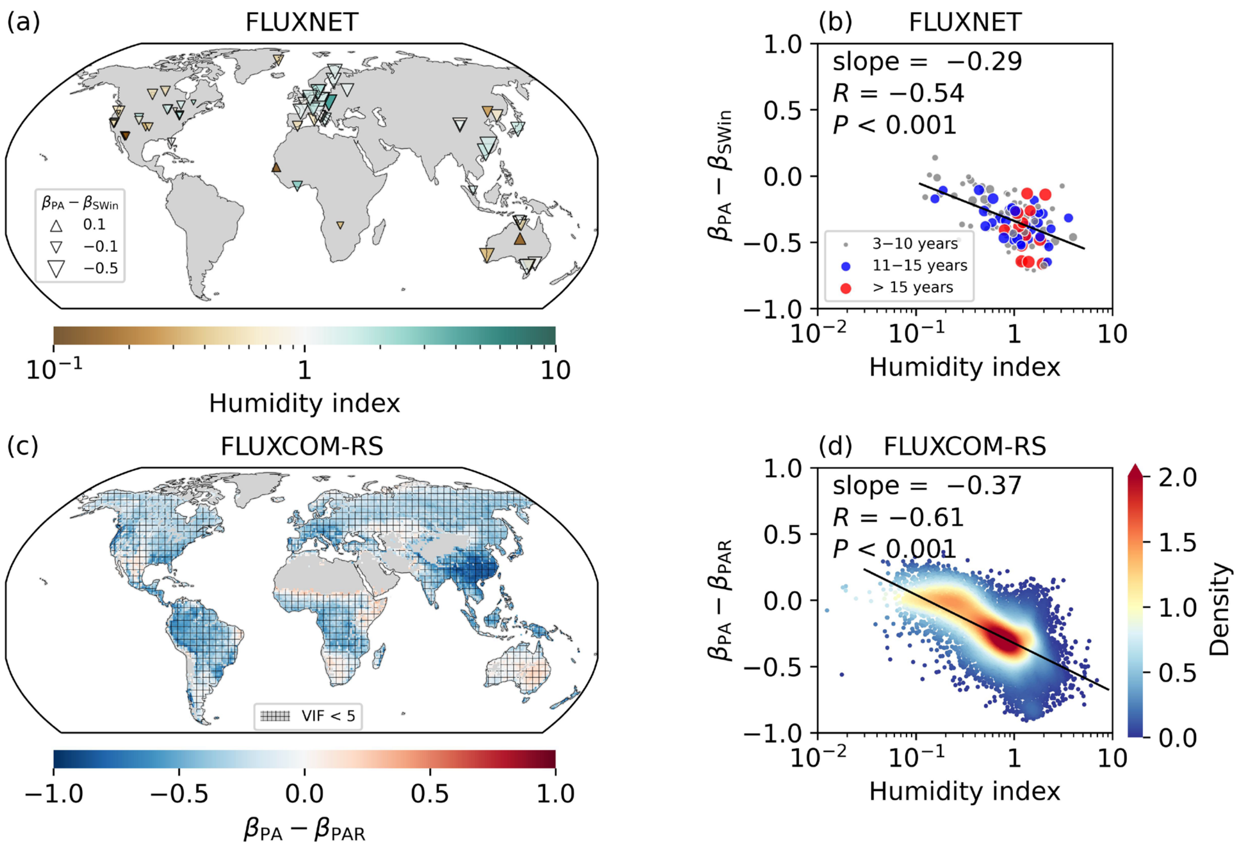

Supplementary Figure 7. Humidity index (HI) spatially shapes the difference between partial sensitivity of standardized gross primary productivity (GPP*) to standardized precipitation amount (PA*) ($\boldsymbol{\beta_{PA}}$) and GPP* to photosynthetically active radiation (PAR*) ($\boldsymbol{\beta_{PAR}}$), represented as ($\boldsymbol{\beta_{PA} - \beta_{PAR}}$) (equation (9), Methods). As in Fig. 1 but for $\beta_{PA} - \beta_{PAR}$ rather than GPP*-to-CF* sensitivity. Since there are only two regressors in equation (9), the variance inflation factors (VIFs) are the same for both regressors. The grid-hatched areas in (**c**) indicate regions where the VIF is less than 5.

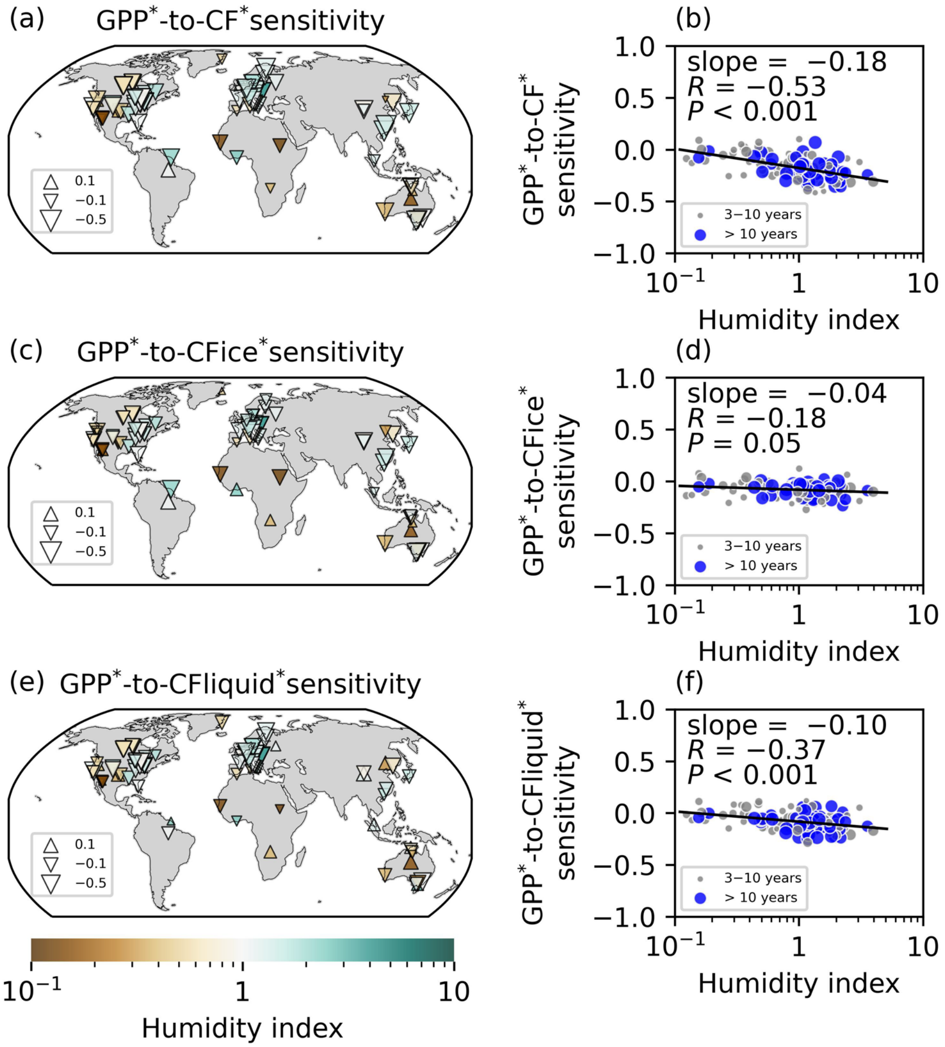

Supplementary Figure 8. Humidity index (HI) spatially shapes the sensitivity of gross primary productivity (GPP) to cloud fraction (CF) across global ecosystems, derived from GPP data from FLUXNET measurements and CF data from the Moderate Resolution Imaging Spectroradiometer (MODIS) onboard Terra. (**a**) and (**b**) As in Fig. 1a and b but using MODIS/Terra-matched CF. (**c**) and (**d**) As in (**a**) and (**b**) but for the retrieved single-layer ice cloud fraction (CFice). (**e**) and (**f**) As in (**a**) and (**b**) but for the retrieved single-layer liquid cloud fraction (CFliquid).

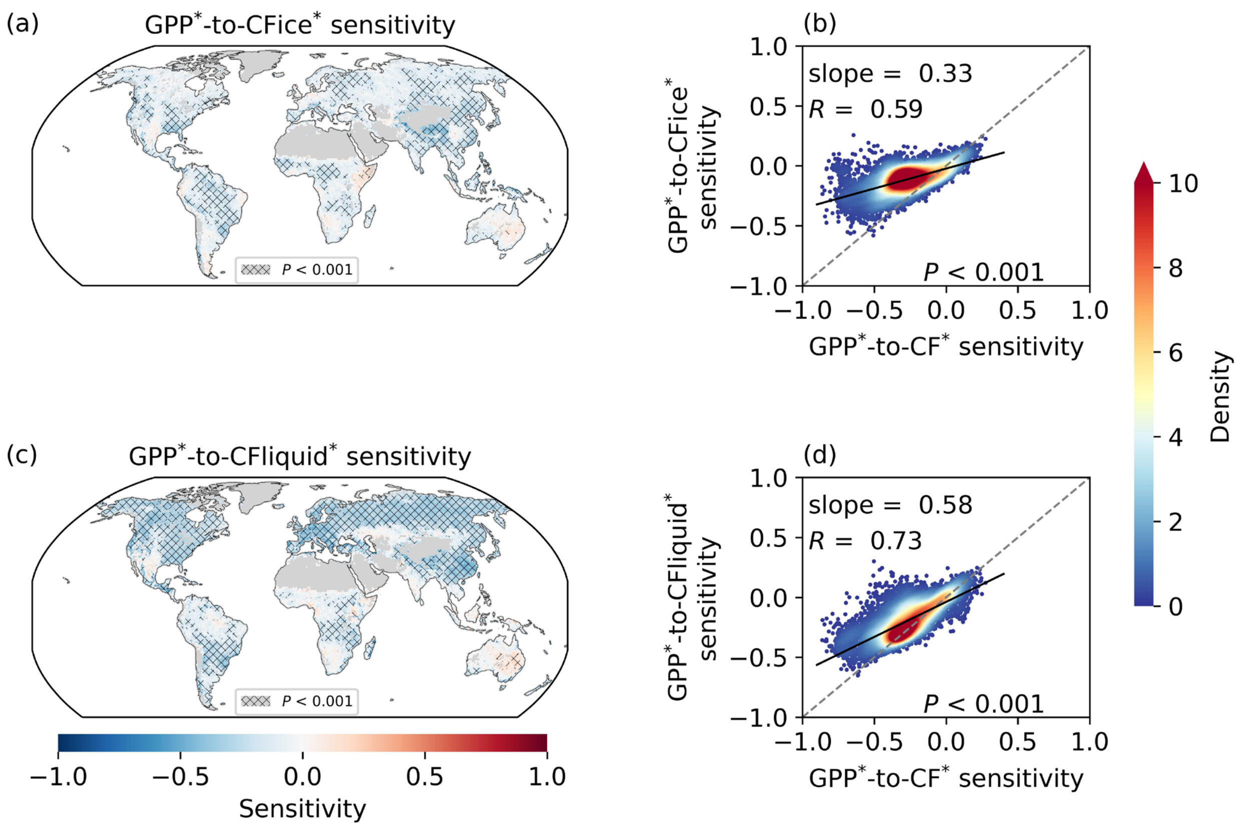

Supplementary Figure 9. Sensitivities of gross primary productivity (GPP) to cloud fraction (CF) for different cloud phases. (**a**) As Fig. 1c but for single-layer ice cloud fraction (CFice). (**b**) Comparison between GPP*-to-CFice* sensitivity and GPP*-to-CF* sensitivity across grids in (**a**). (**c**) and (**d**) As in (**a** and **b**) but for single-layer liquid cloud fraction (CFliquid). All comparisons against GPP*-to-CF* sensitivity are presented as density plots across grids, with the colour bar showing the Gaussian kernel density estimate. The linear regressions are represented by the black lines, with the texts displaying the slope of the linear fit, correlation coefficient (*R*), and *P*-value from a Student's t test. The 1:1 line in each density subplot is marked with a grey dotted line.

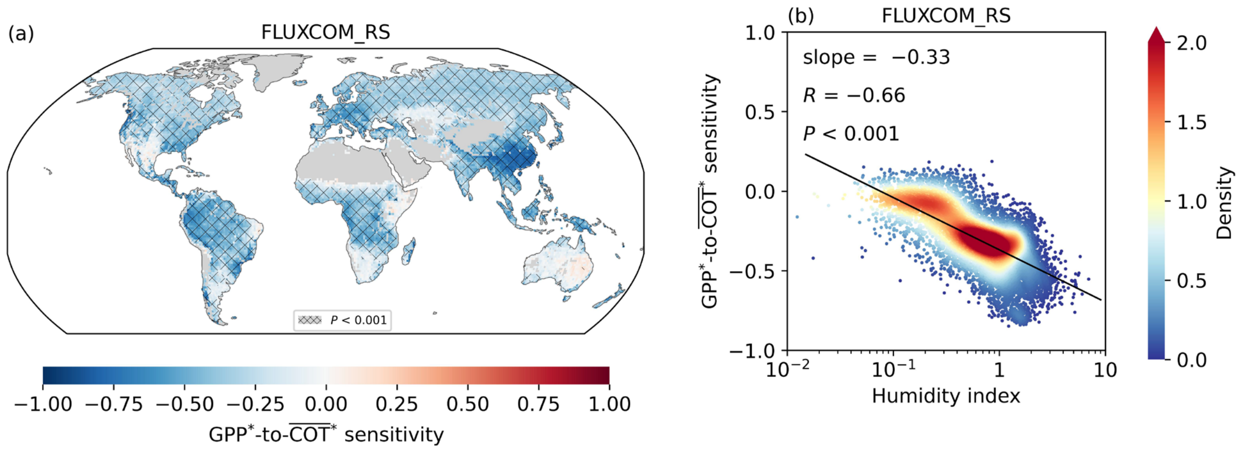

Supplementary Figure 10. Humidity index (HI) spatially shapes the sensitivity of gross primary productivity (GPP) to grid-mean cloud optical thickness ($\overline{\text{COT}}$) across global ecosystems. As in Fig. 1c and d but for the GPP*-to-$\overline{\text{COT}}$* sensitivity.

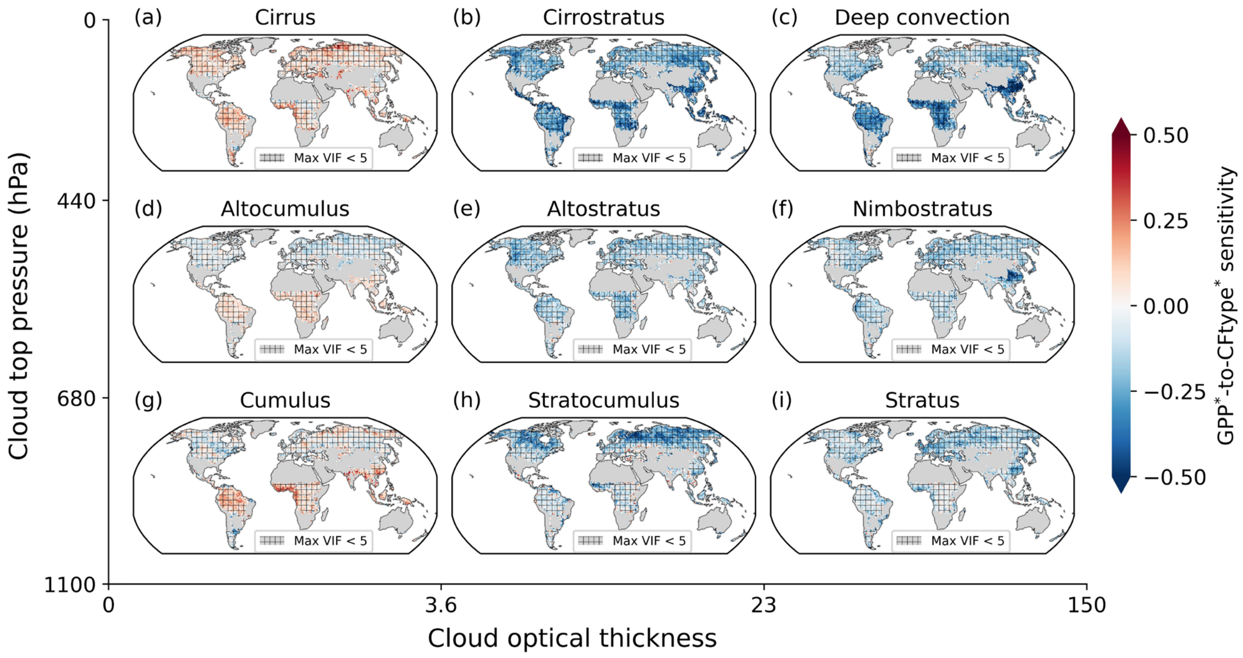

Supplementary Figure 11. Partial sensitivities of gross primary productivity (GPP) to cloud fraction (CF) for nine cloud types (equation (5), Methods). Map of the partial sensitivity of GPP* to the CF* of (**a**) cirrus, (**b**) cirrostratus, (**c**) deep convection, (**d**) altocumulus, (**e**) altostratus, (**f**) nimbostratus, (**g**) cumulus, (**h**) stratocumulus, and (**i**) stratus, respectively. The 8-daily GPP data are from FLUXCOM-RS. The 8-daily CF data for nine cloud types are derived from the joint histograms of cloud optical thickness (COT) and cloud top pressure (CTP), classified according to the International Satellite Cloud Climatology Project (ISCCP) classification scheme, using data from the Moderate Resolution Imaging Spectroradiometer (MODIS) aboard Terra. Non-vegetated areas are masked (Methods). The grid-hatched areas indicate regions where the maximum variance inflation factor (VIF) is less than 5.

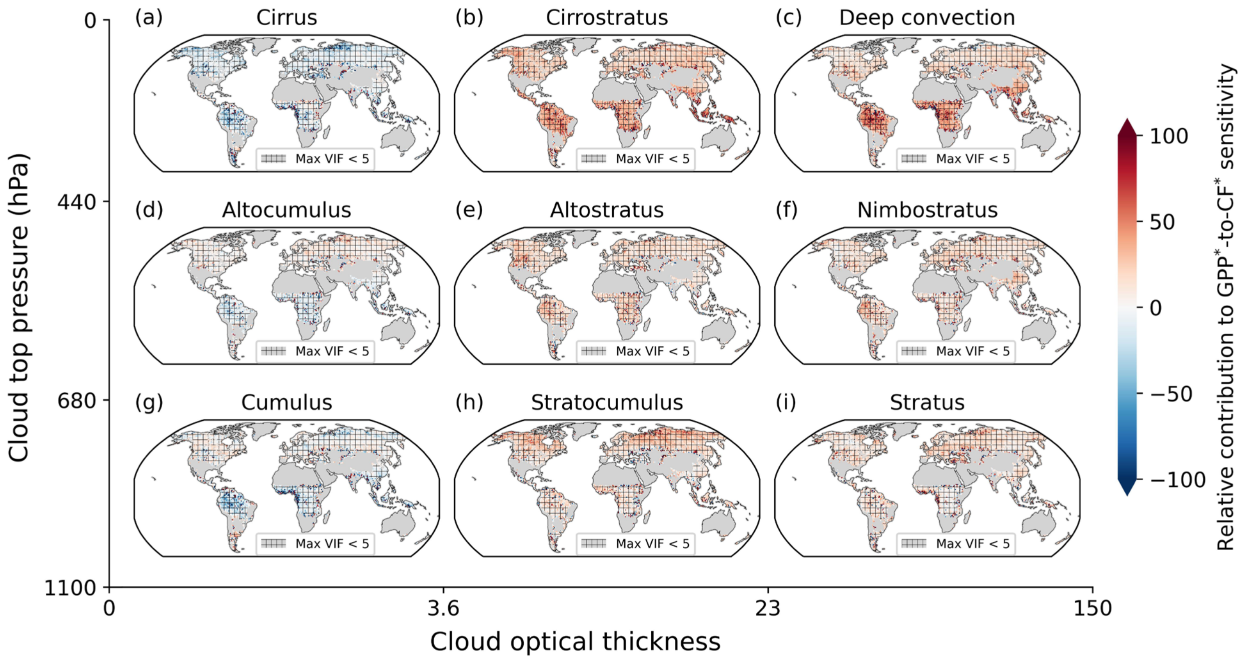

Supplementary Figure 12. Relative contributions of the nine cloud types to the sensitivity of gross primary productivity (GPP) to total cloud fraction (CF) (equation (6), Methods). As in Supplementary Fig. 11, but for the relative contributions of the nine cloud types to GPP^*-to-CF^* sensitivity.

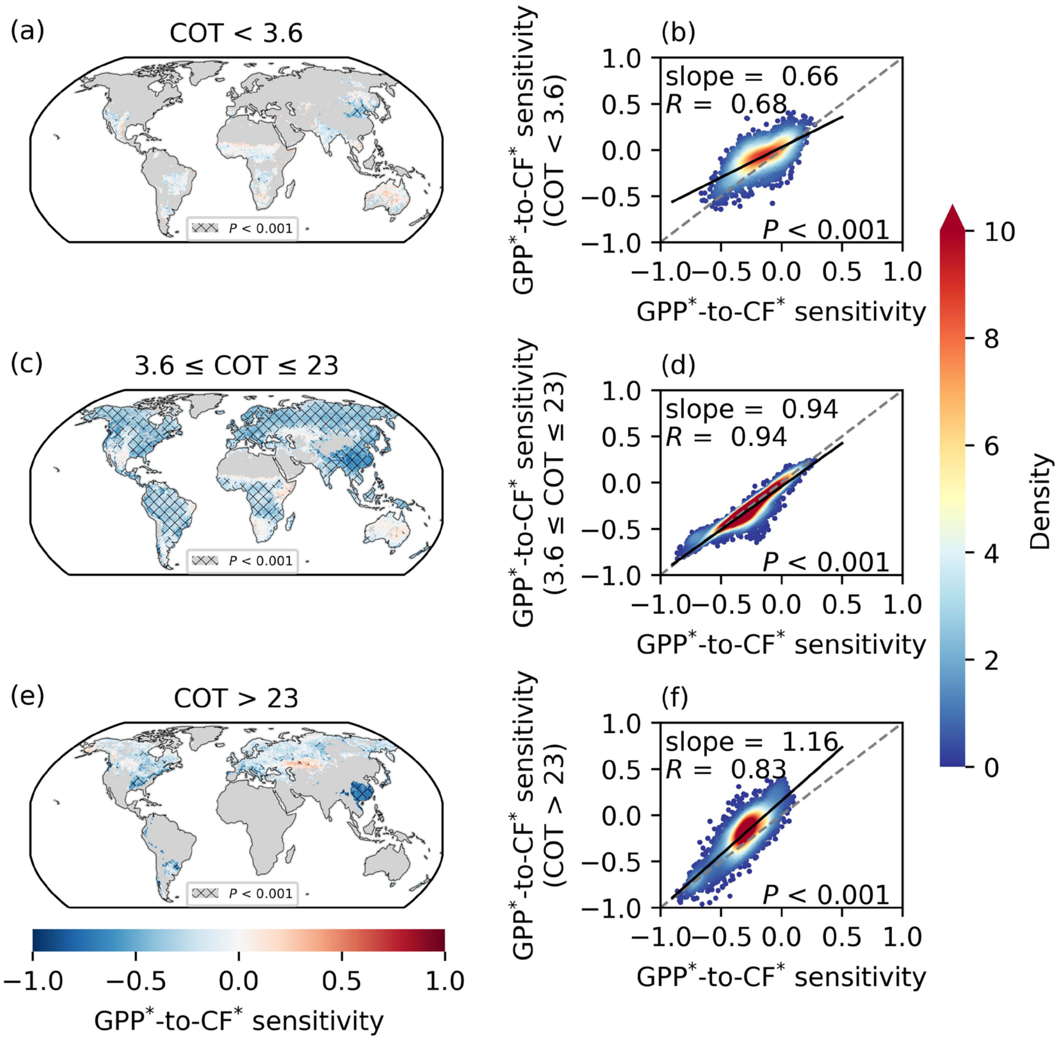

Supplementary Figure 13. Sensitivities of gross primary productivity (GPP) to cloud fraction (CF) for different cloud optical depth (COT). (**a**) As in Fig. 1c but for within-cloud COT < 3.6. (**b**) Comparison between GPP*-to-CF* sensitivity with COT < 3.6 and GPP*-to-CF* sensitivity across grids in (**a**). (**c**) and (**d**) As in (**a**) and (**b**) but for within-cloud 3.6 ≤ COT ≤ 23. (**e**) and (**f**) As in (**a**) and (**b**) but for within-cloud COT > 23. All comparisons against GPP*-to-CF* sensitivity are presented as density plots across grids, with the colour bar showing the Gaussian kernel density estimate. Only grids with a sample number greater than 100 are included in this analysis. The linear regressions are represented by the black lines, with the texts displaying the slope of the linear fit, correlation coefficient (*R*), and *P*-value from a Student's t test. The 1:1 line in each density subplot is marked with a grey dotted line.

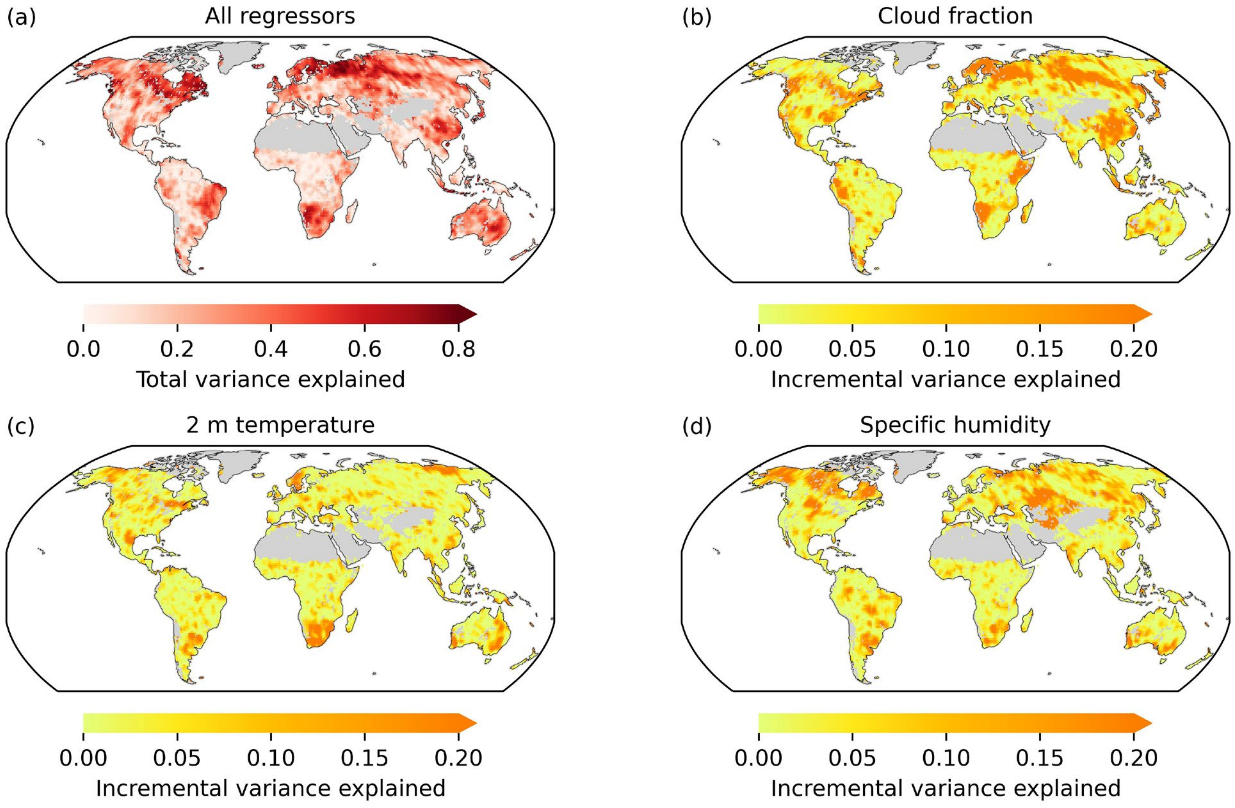

Supplementary Figure 14. Total variance explained of the regressors in equation (7) and incremental variance explained of each regressor. (**a**) The total variance explained of the three regressors in equation (7). The incremental variance explained (Methods) of (**b**) cloud fraction, (**c**) 2 m temperature, and (**d**) specific humidity, respectively.

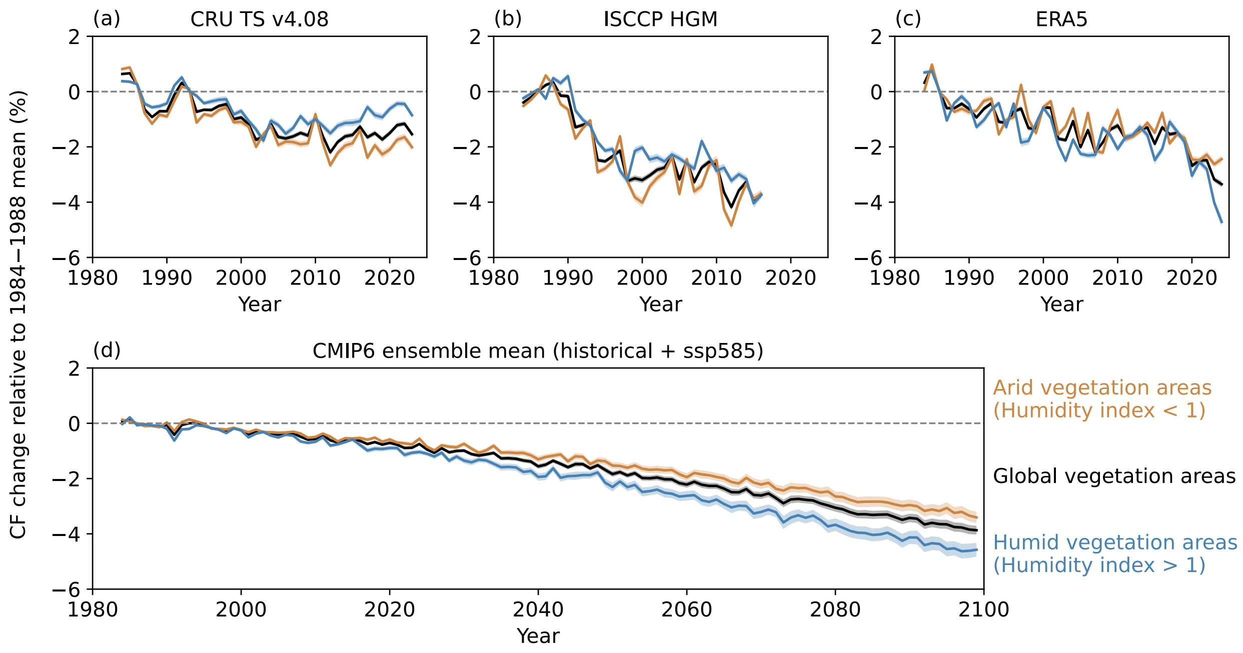

Supplementary Figure 15. Long-term changes in cloud fraction (CF). As in Fig. 4 but for CF.

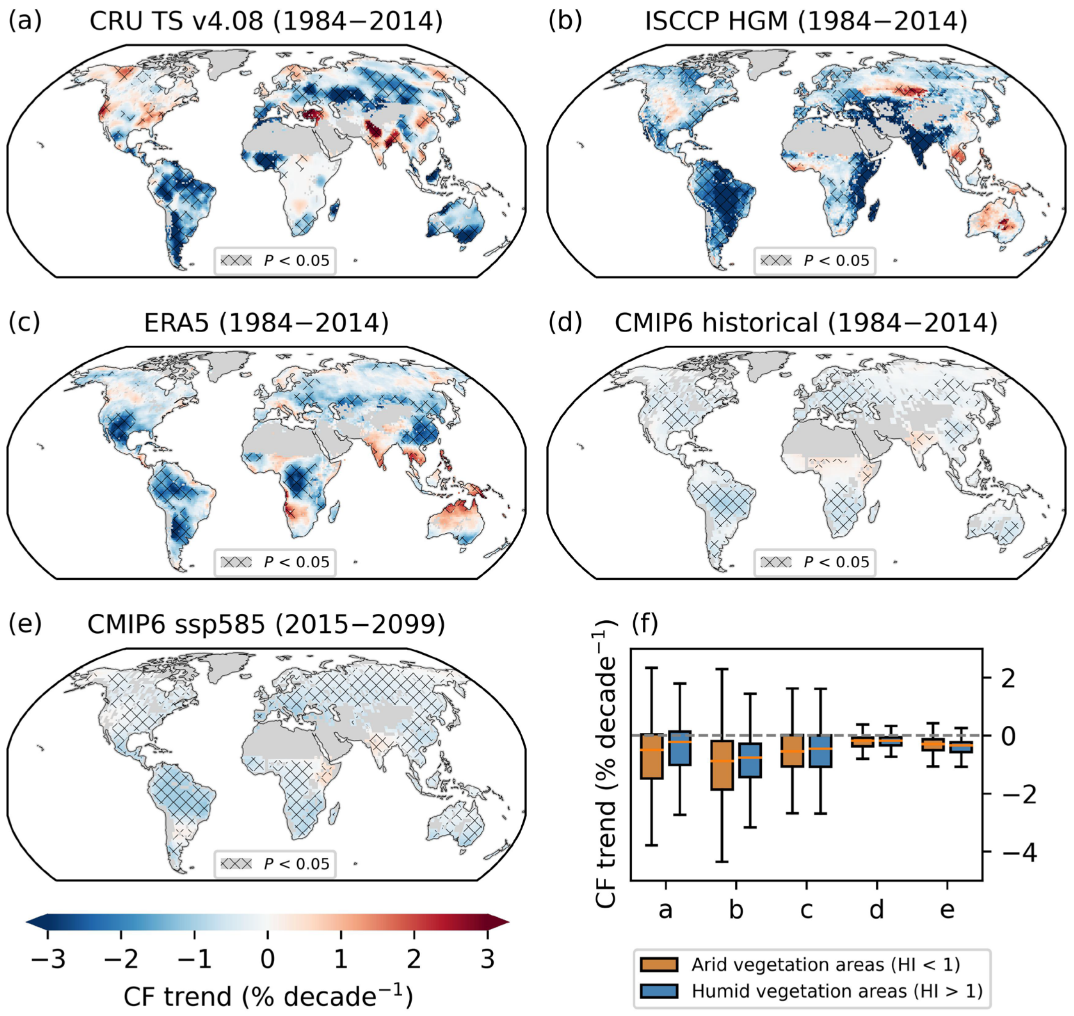

Supplementary Figure 16. Long-term trends in cloud fraction (CF). Map of linear trend in CF using data from (**a**) the Climatic Research Unit Time-Series Version 4.08 (CRU TS v4.08) between 1984 and 2014, (**b**) the International Satellite Cloud Climatology Project High-Resolution Global Monthly product (ISCCP-HGM) between 1984 and 2014, (**c**) the European Centre for Medium-Range Weather Forecasts (ECMWF) fifth generation reanalysis (ERA5) between 1984 and 2014, the sixth phase Coupled Model Intercomparison Project (CMIP6) 39 models ensemble mean for (**d**) historical (1984–2014) and (**e**) "ssp585" projected (2015–2099) periods. The cross-hatched areas represent regions where *P*-value from a Student's t-test is less than 0.05. (**f**) Box plots for the trends in (**a**−**e**) for arid vegetation areas with humidity index (HI) < 1 and humid vegetation areas with HI > 1. The box extends from the first quartile (Q1) to the third quartile (Q3) of the data, with an orange line at the median. The whiskers extend from the box to the farthest data point lying within 1.5 times the inter-quartile range (IQR) from the box.

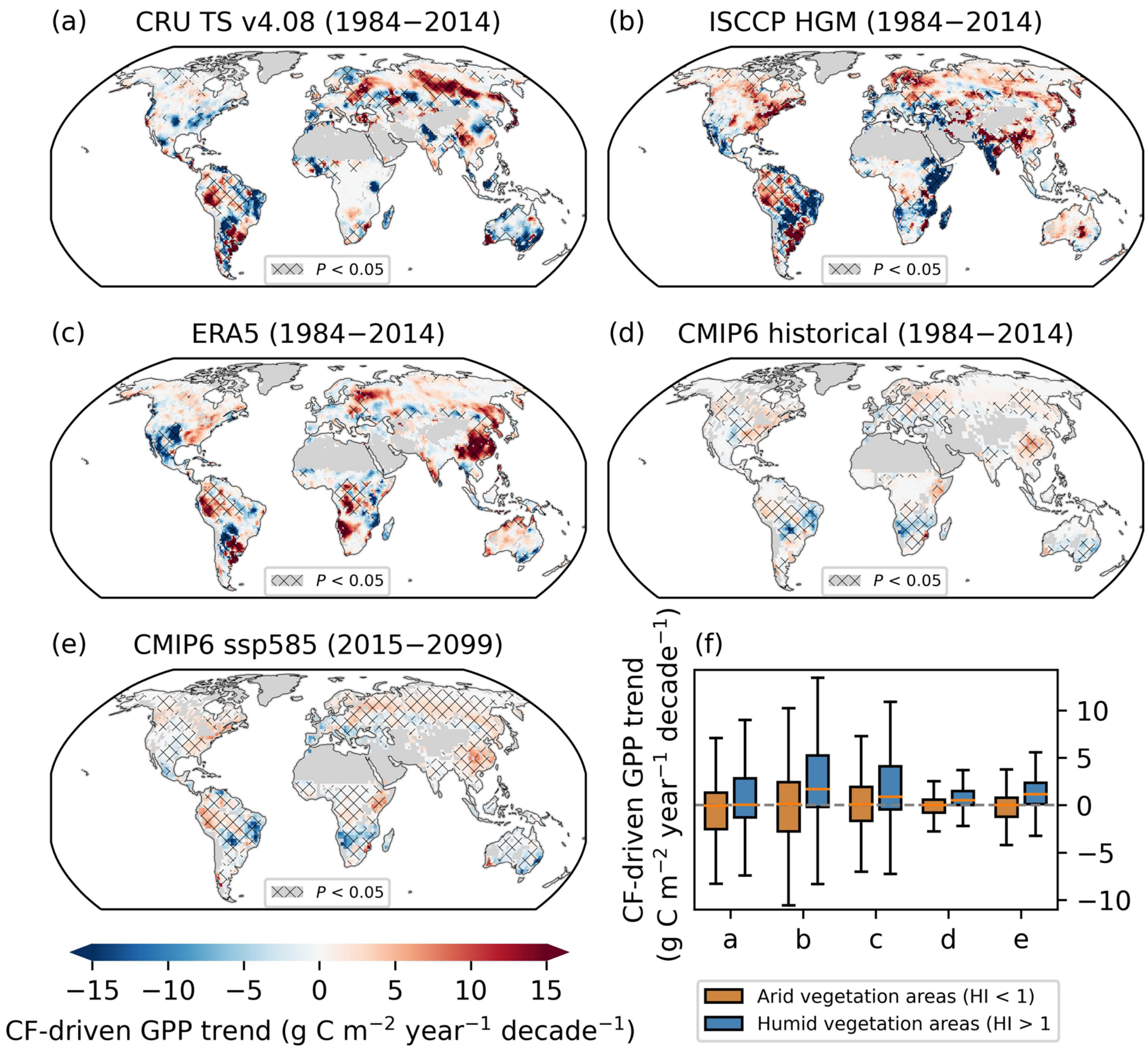

Supplementary Figure 17. Long-term trends in gross primary productivity (GPP) driven by cloud fraction (CF). As in Supplementary Fig. 16 but for estimated linear trends in GPP caused by changes in CF (equation (12), Methods). The annual scale GPP-to-CF sensitivity used for the estimations is derived from Fig. 3a.

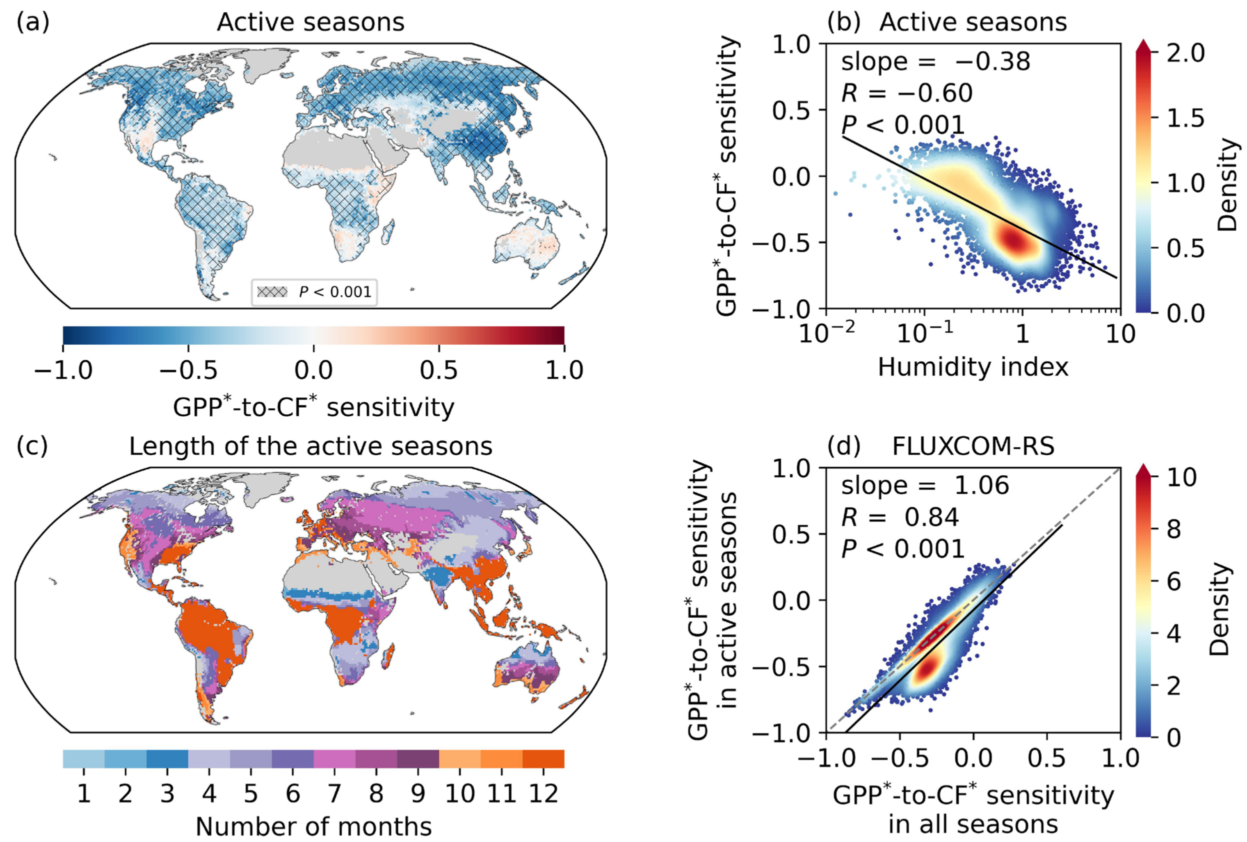

Supplementary Figure 18. Humidity index (HI) spatially shapes the sensitivity of gross primary productivity (GPP) to cloud fraction (CF) across global ecosystems in meteorological active seasons (Methods). (**a**) and (**b**) As in Fig. 1c and d but only for meteorological active seasons. (**c**) Map of the length of the meteorological active seasons. (**d**) Density plot showing the comparison between GPP*-to-CF* sensitivity in the meteorological active seasons and in all seasons across grids. The colour bars showing the Gaussian kernel density estimates. The linear regressions are represented by the black lines, with the texts displaying the slope of the linear fit, correlation coefficient (R), and P-value from a Student's t test. The 1:1 line is marked with a grey dotted line.

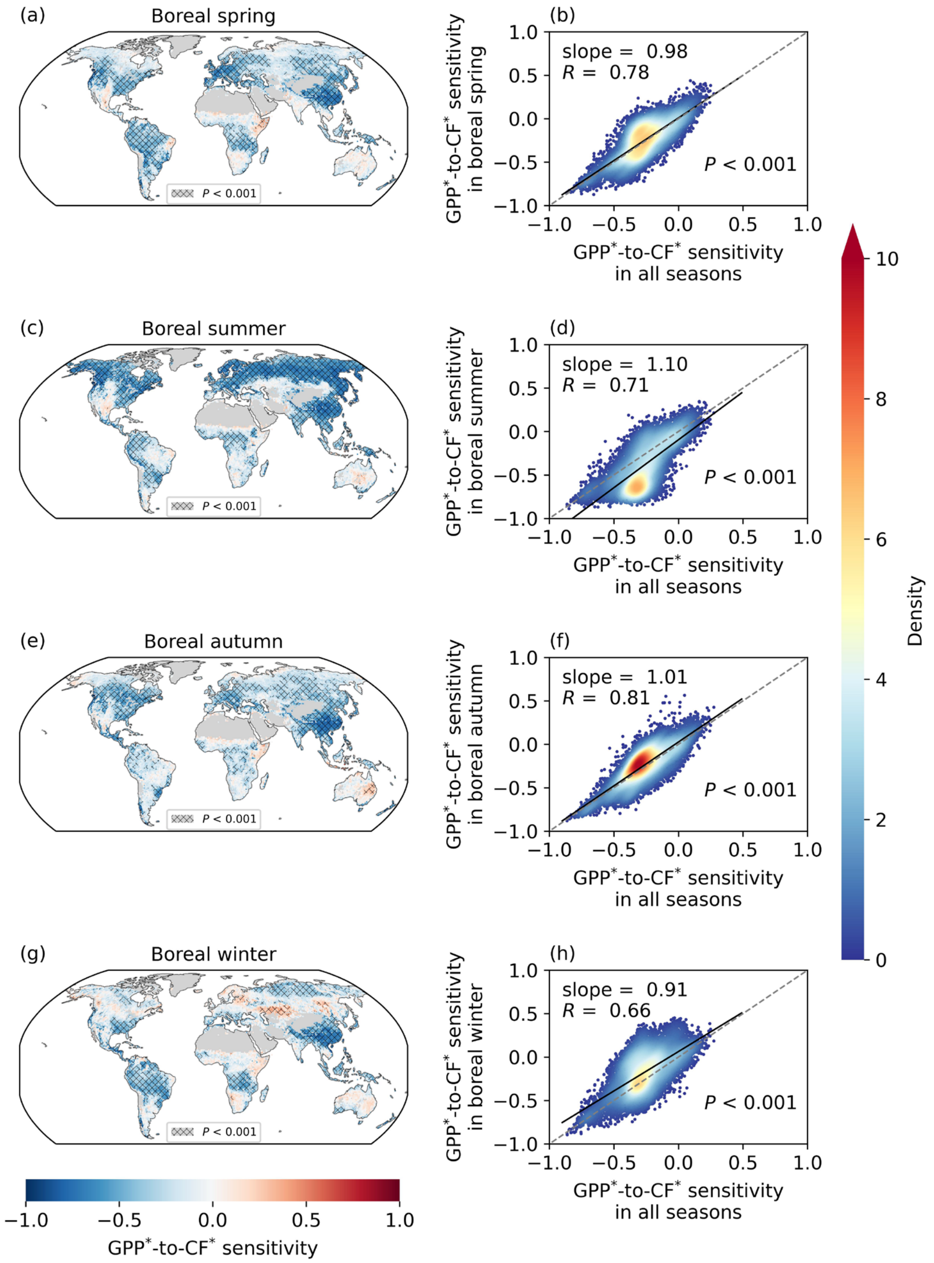

Supplementary Figure 19. Humidity index (HI) spatially shapes the sensitivity of gross primary productivity (GPP) to cloud fraction (CF) across global ecosystems in different seasons. (**a**) As in Fig. 1c but only for the boreal spring (March, April, and May). (**b**) Density plot showing the comparison between GPP^*-to-CF^* sensitivity in boreal spring and in all seasons across grids. (**c**) and (**d**) As is (**a**) and (**b**) but for the boreal summer (June, July, and August). (**e**) and (**f**) As is (**a**) and (**b**) but for the boreal autumn (September, October, and November). (**g**) and (**h**) As is (**a**) and (**b**) but for the boreal winter (December, January, and February).

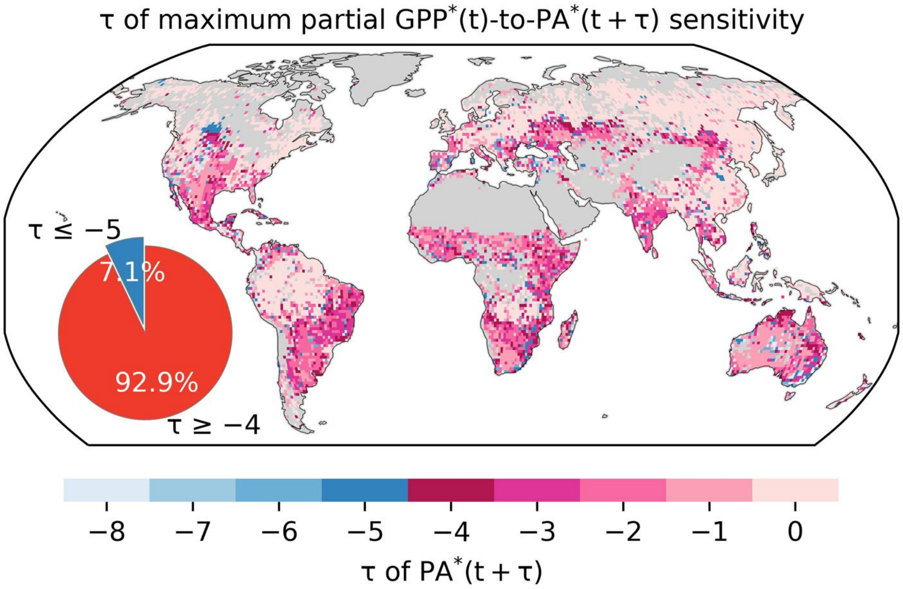

Supplementary Figure 20. Effective response period of gross primary productivity (GPP) to precipitation amount (PA). Map of lag time τ for the maximum partial $\mathrm{GPP}^*(t)$-to-$\mathrm{PA}^*(t+\tau)$ sensitivity (equation (10), Methods). Only pixels with a statistically significant sensitivity (P-value < 0.001) are shown. The pie chart in the lower-left corner of the map illustrates the proportion of pixels with $\tau \leq -5$ and $\tau \geq -4$ for the maximum partial $\mathrm{GPP}^*(t)$-to-$\mathrm{PA}^*(t+\tau)$ sensitivity. Note the unit of τ is an 8-day interval.

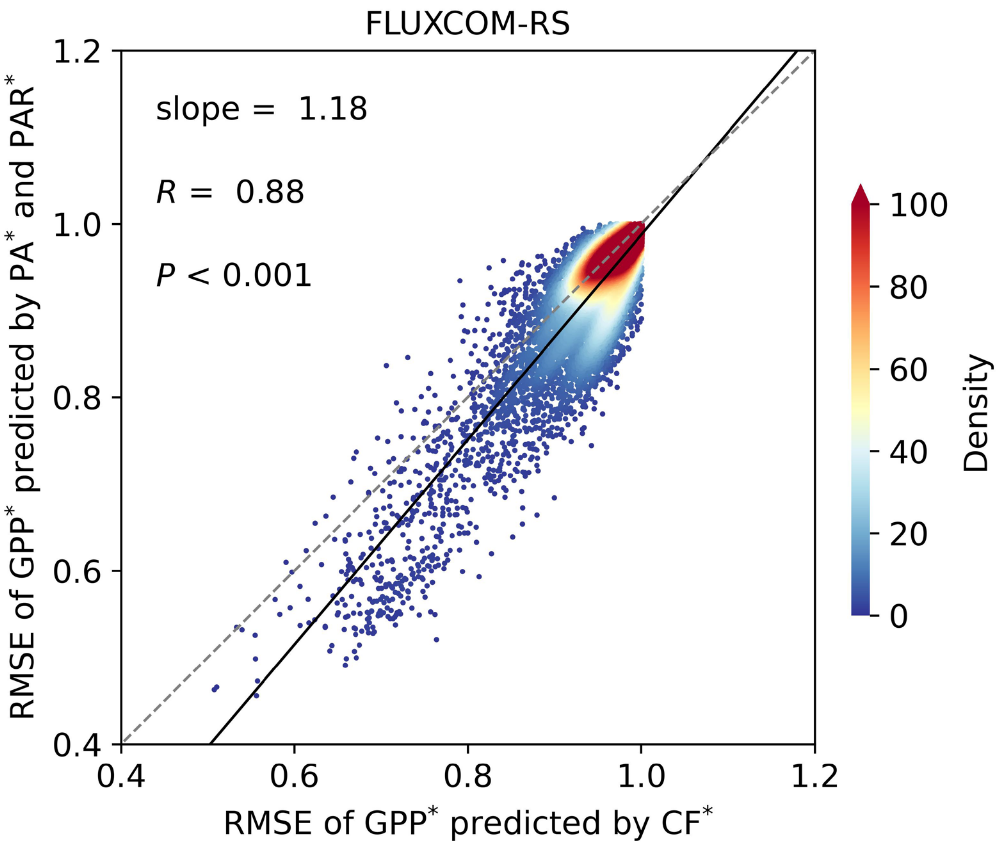

Supplementary Figure 21. Comparison of root mean squared error (RMSE) when predicting gross primary productivity (GPP) using single (cloud fraction (CF)) and double (precipitation amount (PA) and photosynthetically active radiation (PAR)) regressors. Density plot of RMSE of GPP^* predicted by CF^* against RMSE of GPP^* predicted by PA^* and PAR^* across vegetation grids. The colour bar shows the Gaussian kernel density estimate. The analysis is based on the periods between 2001 and 2020 using 8-daily GPP from FLUXCOM-RS, CF from the Moderate Resolution Imaging Spectroradiometer (MODIS) onboard Terra, PA from the Global Precipitation Measurement (GPM) Integrated Multi-satellitE Retrievals for GPM (IMERG), and photosynthetically active radiation (PAR) from the Clouds and the Earth's Radiant Energy System (CERES). The linear regressions are represented by the black lines, with the texts displaying the slope of the linear fit, correlation coefficient (R), and P-value from a Student's t test. The 1:1 line in each subplot is marked with a grey dotted line.

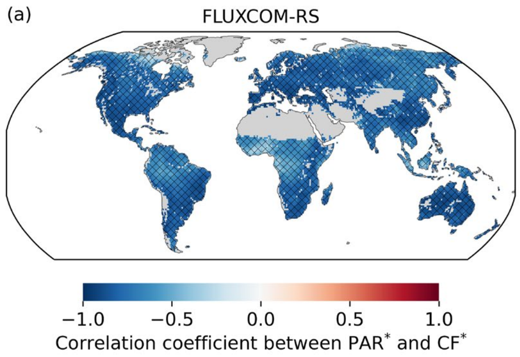

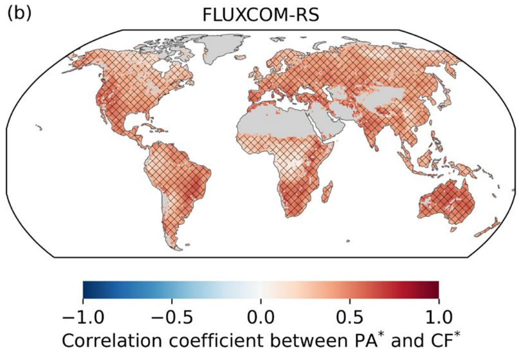

Supplementary Figure 22. Correlations between photosynthetically active radiation (PAR) and cloud fraction (CF), as well as between precipitation amount (PA) and CF. (**a**) Map of the correlation coefficient between PAR^* and CF^*. (**b**) Map of the correlation coefficient between PA^* and CF^*. The analysis is based on the periods between 2001 and 2020 using 8-daily CF from the Moderate Resolution Imaging Spectroradiometer (MODIS) onboard Terra, PA from the Global Precipitation Measurement (GPM) Integrated Multi-satellitE Retrievals for GPM (IMERG), and photosynthetically active radiation (PAR) from the Clouds and the Earth's Radiant Energy System (CERES). The cross-hatched areas represent regions where the *P*-value from a Student's t-test is less than 0.001.

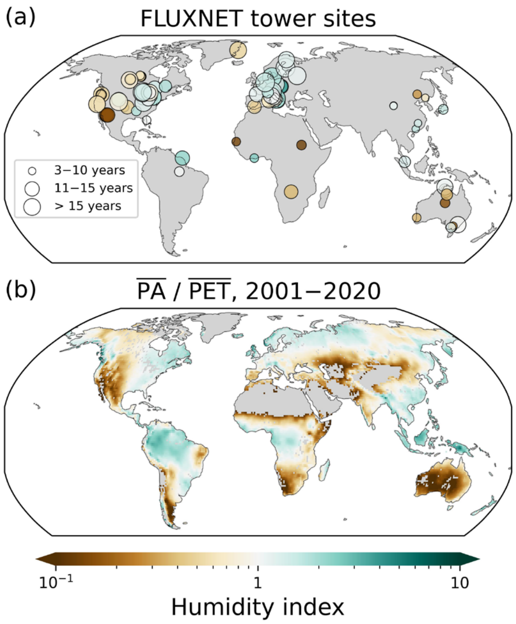

Supplementary Figure 23. FLUXNET tower sites and hydroclimate conditions. (**a**) 114 candidate sites selected for this study, with locations marked with circles. The larger the circle, the longer the measurement record of the site. The colour filling inside each circle corresponds to the humidity index (HI) observed during the measurement period at each site. (**b**) Map of HI between 2001 and 2020 calculated based on data from the Climatic Research Unit Time-Series Version 4.08 (CRU TS v4.08). Non-vegetated areas are masked (Methods).

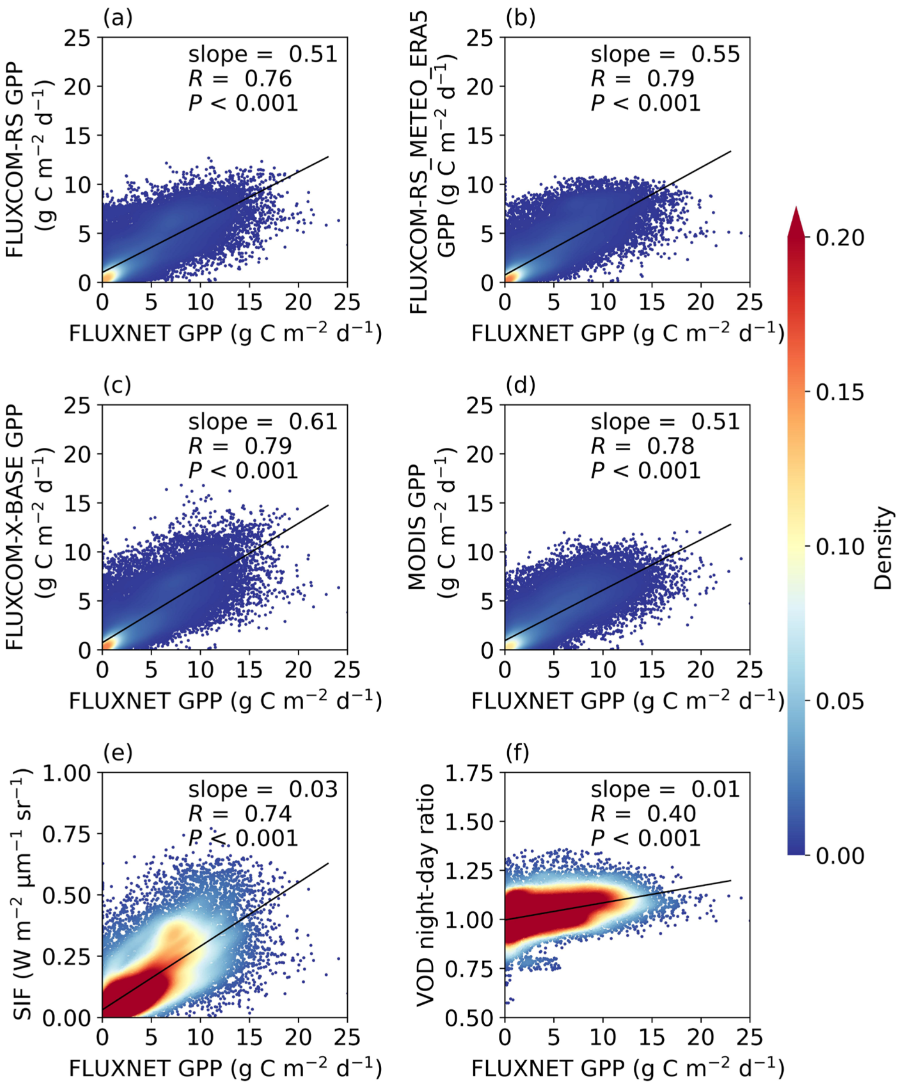

Supplementary Figure 24. Validations of different vegetation indices against gross primary productivity (GPP) measured by FLUXNET. (a–f) Density plot comparing GPP from FLUXCOM-RS, GPP from FLUXCOM-RS_METEO_ERA5, GPP from FLUXCOM-X-BASE, GPP from the Moderate Resolution Imaging Spectroradiometer (MODIS) onboard Terra, solar-induced fluorescence (SIF) from the global dataset of solar-induced chlorophyll fluorescence (GOSIF), and vegetation optical depth (VOD) night-to-day ratio (VODndr) from the Version 3 global land parameter data record (LPDR v3) X-band VOD dataset, respectively, against FLUXNET GPP. The validations are based on 8-daily averages and negative FLUXNET GPP values are removed. The linear regressions are represented by the black lines, with the texts displaying the slope of the linear fit, correlation coefficient (*R*), and *P*-value from a Student's t test. The colour bar shows the Gaussian kernel density estimate.

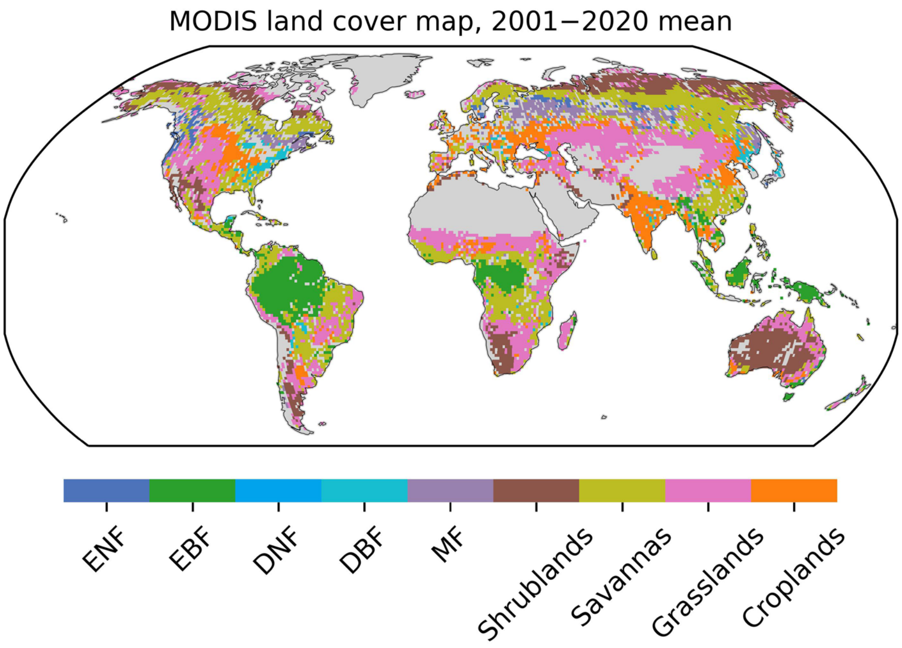

Supplementary Figure 25. Map of the land cover types. The land cover types are derived from the Terra and Aqua combined Moderate Resolution Imaging Spectroradiometer (MODIS) land cover product (MCD12C1, Version 6.1) with the International Geosphere-Biosphere Programme (IGBP) classification scheme (Methods). ENF, EBF, DNF, DBF, and MF are, respectively, evergreen needleleaf forest, evergreen broadleaf forest, deciduous needleleaf forest, deciduous broadleaf forest, and mixed forest.

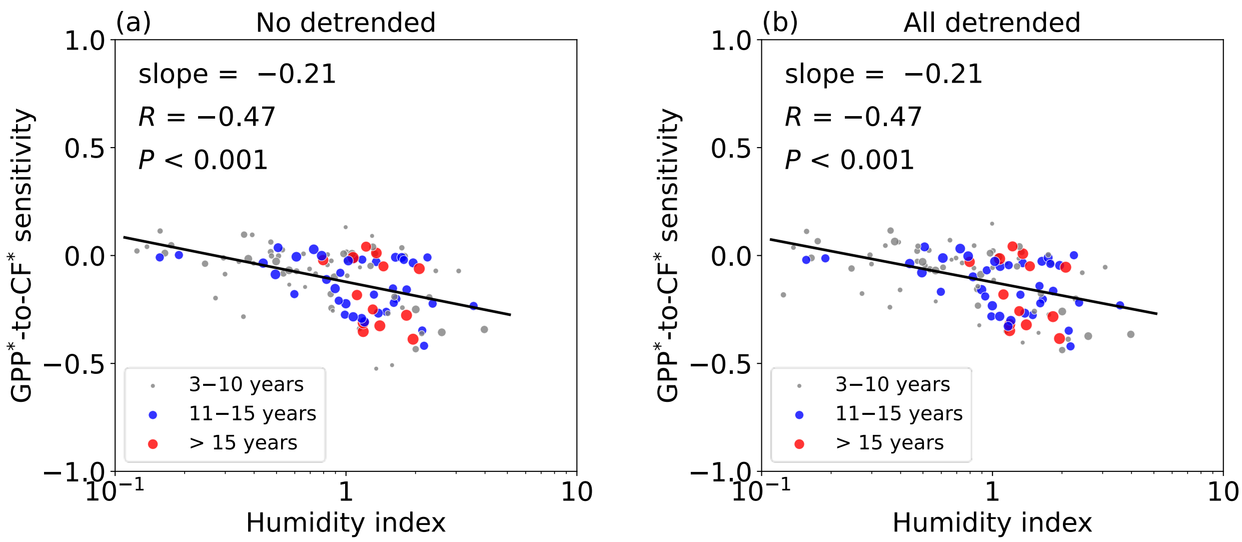

Supplementary Figure 26. Tests with the impacts of detrending in the processing of FLUXNET data. As in Fig. 1b but (**a**) without detrending and (**b**) data at all the sites are detrended.

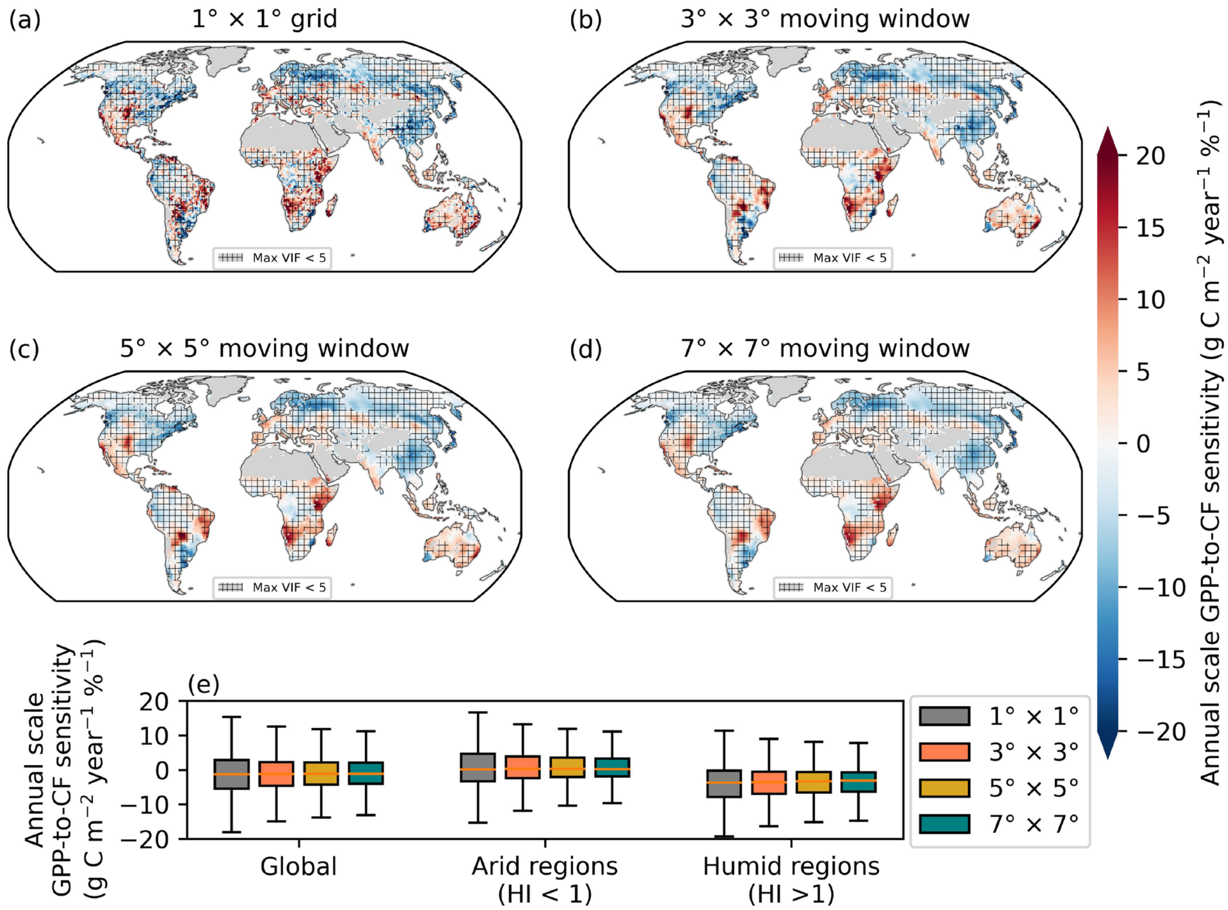

Supplementary Figure 27. The annual scale GPP-to-CF sensitivity holds across different spatial aggregation approaches. As in Fig. 3c but with (**a**) no spatial aggregation, (**c**) 5° × 5° moving window, and (**d**) 7° × 7° moving window. Fig. 3c is processed based on a 3° × 3° moving window, which is replotted in (**b**). (**e**) Box plots of the data in (**a**−**d**) with three categories labeled: global vegetation areas, arid vegetation areas with humidity index (HI) < 1, and humid vegetation areas with HI > 1. The box extends from the first quartile (Q1) to the third quartile (Q3) of the data, with an orange line at the median. The whiskers extend from the box to the farthest data point lying within 1.5 times the inter-quartile range (IQR) from the box.

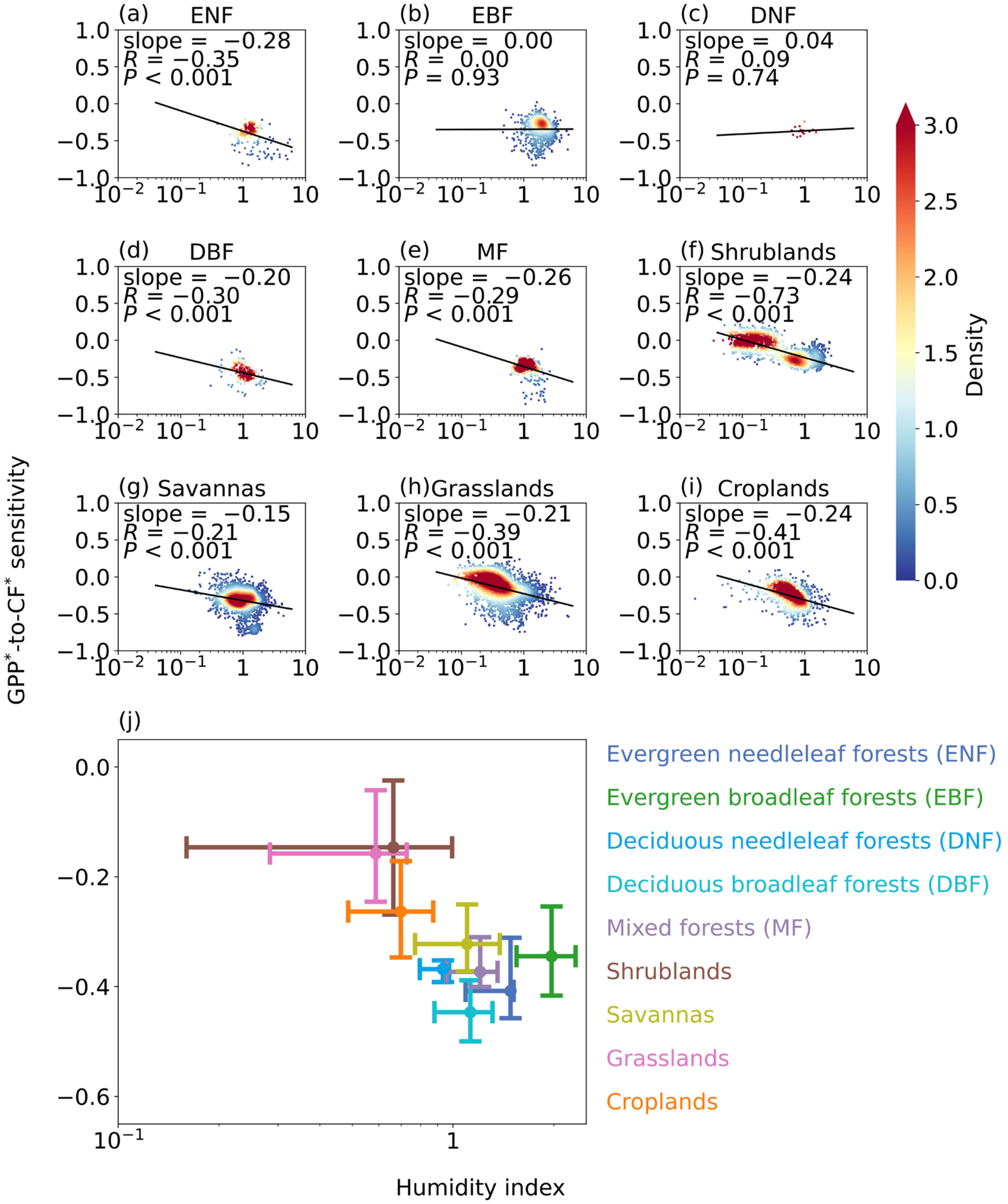

Supplementary Figure 28. Sensitivities of gross primary productivity (GPP) to cloud fraction (CF) across different land covers and their relationships with humidity index (HI). (**a**−**i**) As in Fig. 1d but for regions classified as evergreen needleleaf forest, evergreen broadleaf forest, deciduous needleleaf forest, deciduous broadleaf forest, mixed forest, shrublands, savannas, grasslands, and croplands, respectively. (**j**) Comparison of GPP*-to-CF* sensitivity and HI across different land cover types. Points represent area-weighted averages, with error bars indicating the first and third quartiles.

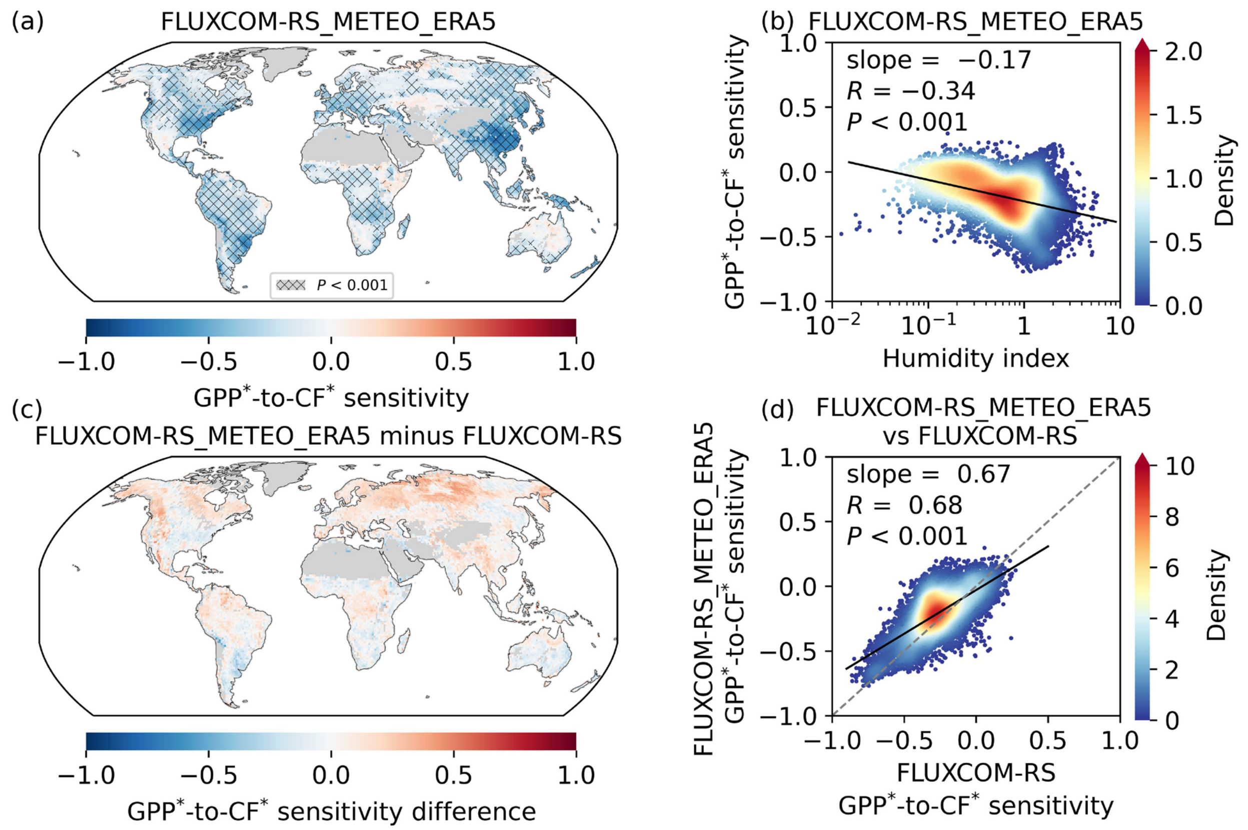

Supplementary Figure 29. Humidity index (HI) spatially shapes the sensitivity of gross primary productivity (GPP) to cloud fraction (CF) across global ecosystems, derived from GPP data from FLUXCOM-RS_METEO_ERA5 and CF data from the Moderate Resolution Imaging Spectroradiometer (MODIS) onboard Terra. (**a**) and (**b**) As in Fig. 1c and d but using GPP data from FLUXCOM-RS_METEO_ERA5. (**c**) The difference map between (**a**) and Fig. 1c. (**d**) Density plot showing the comparison between the calculated GPP*-to-CF* sensitivity based on GPP data from FLUXCOM-RS_METEO_ERA5 and FLUXCOM-RS across grids in (**c**). The linear regression is represented by the black line, with the texts displaying the slope of the linear fit, correlation coefficient (*R*), and *P*-value from a Student's t test. The colour bar shows the Gaussian kernel density estimate. The 1:1 line is marked with a grey dotted line.

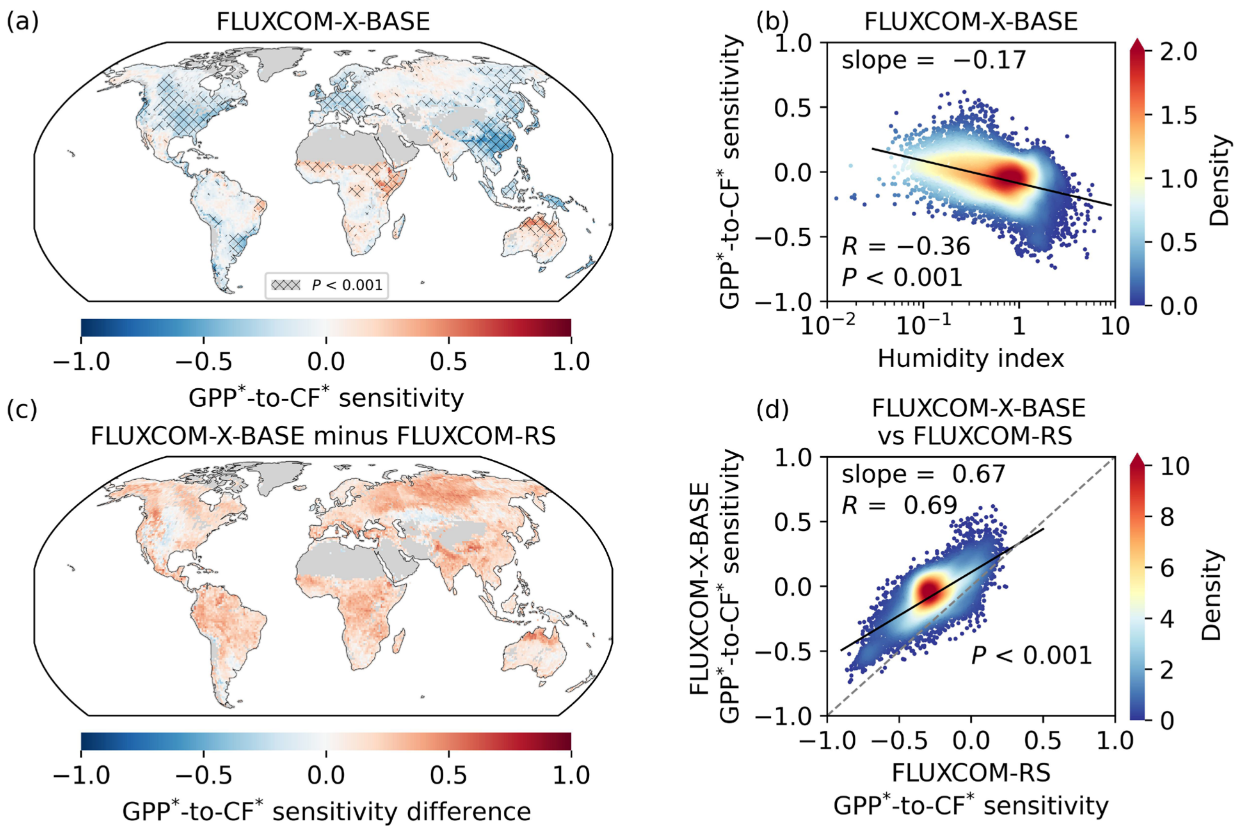

Supplementary Figure 30. Humidity index (HI) spatially shapes the sensitivity of gross primary productivity (GPP) to cloud fraction (CF) across global ecosystems, derived from GPP data from FLUXCOM-X-BASE and CF data from the Moderate Resolution Imaging Spectroradiometer (MODIS) onboard Terra. As in Supplementary Fig. 29 but using GPP data from FLUXCOM-X-BASE.

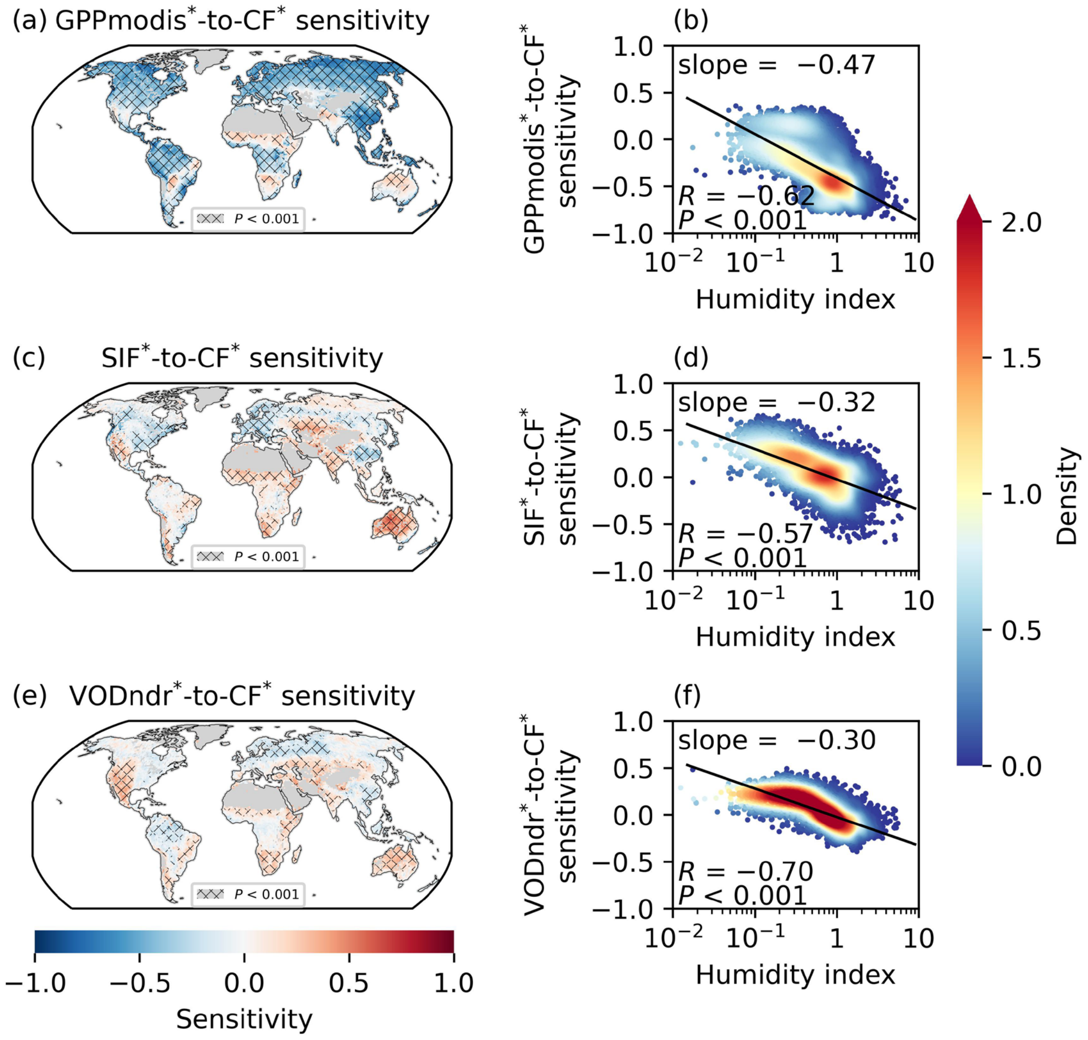

Supplementary Figure 31. Humidity index (HI) spatially shapes the sensitivity of vegetation dynamics to cloud fraction (CF) across global ecosystems, derived from different vegetation indices and CF data from the Moderate Resolution Imaging Spectroradiometer (MODIS) onboard Terra. (**a**) and (**b**) As in Fig. 1c and d but for gross primary productivity (GPP) from MODIS/Terra, and periods between 2001 and 2021. (**c**) and (**d**) As in Fig. 1c and d but for solar-induced fluorescence (SIF) from the global dataset of solar-induced chlorophyll fluorescence (GOSIF), and periods between 2001 and 2021. (**e**) and (**f**) As in Fig. 1c and d but for vegetation optical depth (VOD) night-to-day ratio (VODndr) from the Version 3 global land parameter data record (LPDR v3) X-band VOD dataset, and periods between 2003 and 2021.

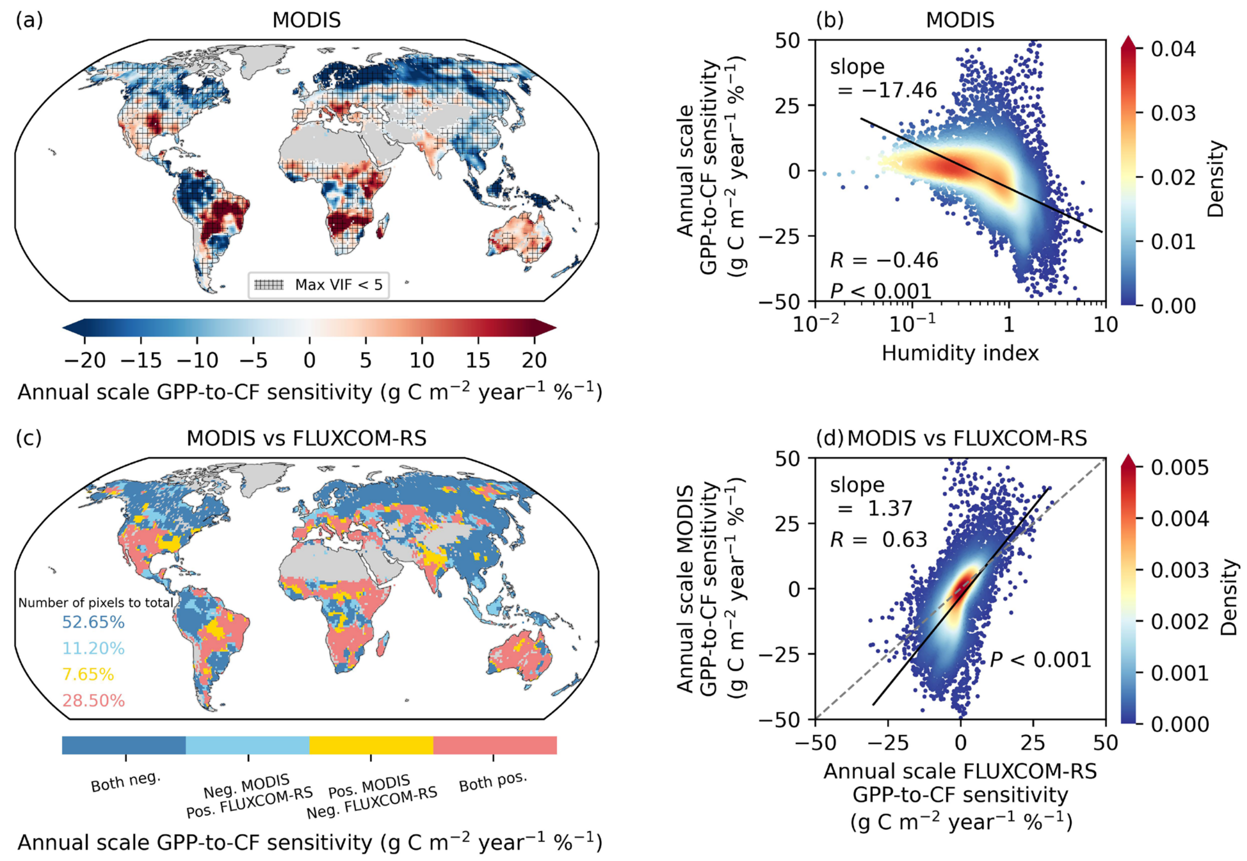

Supplementary Figure 32. Estimated annual scale sensitivity of gross primary productivity (GPP) to cloud fraction (CF), derived from GPP and CF data from the Moderate Resolution Imaging Spectroradiometer (MODIS) onboard Terra. (**a**) and (**b**) As in Fig. 3a and b but for GPP data from MODIS. (**c**) and (**d**) As in Fig. 3c and d but for comparing annual scale GPP-to-CF sensitivities calculated based on GPP data from MODIS and FLUXCOM-RS.

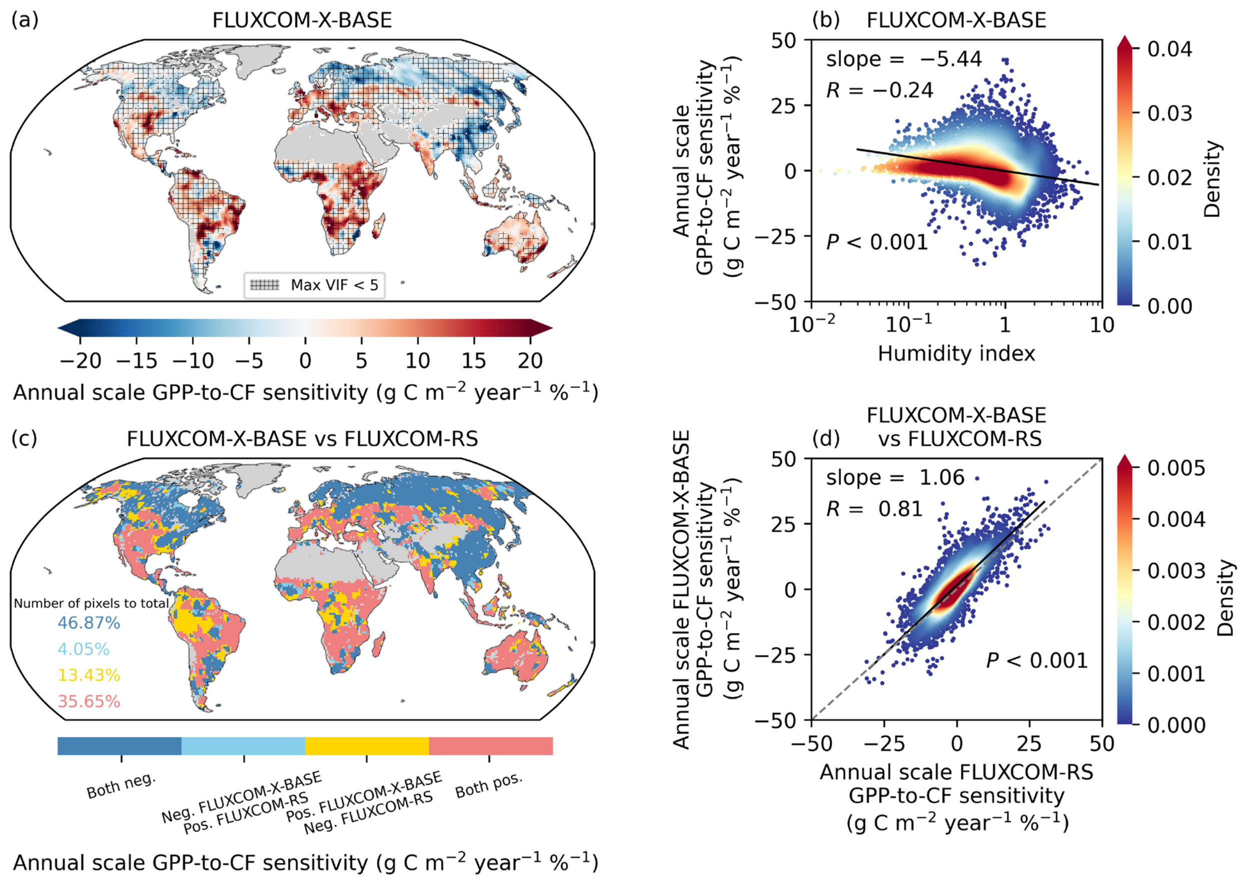

Supplementary Figure 33. Estimated annual scale sensitivity of gross primary productivity (GPP) to cloud fraction (CF), derived from GPP data from FLUXCOM-X-BASE and CF data from the Moderate Resolution Imaging Spectroradiometer (MODIS) onboard Terra. As in Supplementary Fig. 32 but for GPP data from FLUXCOM-X-BASE.

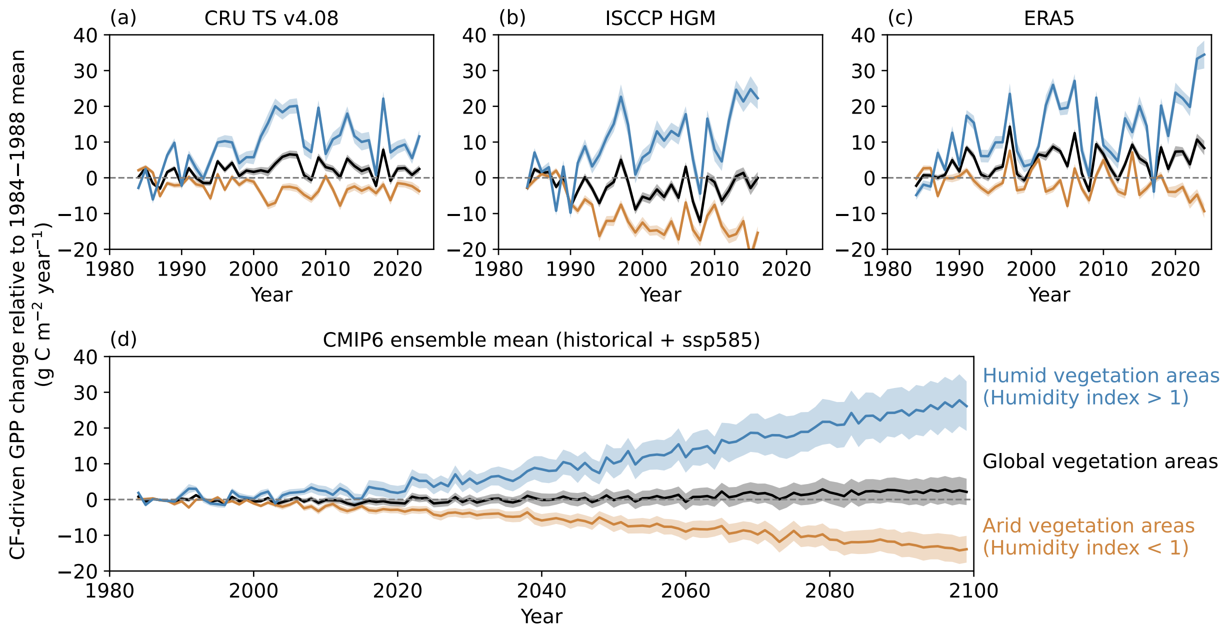

Supplementary Figure 34. Shifts in gross primary productivity (GPP) from arid to humid regions driven by cloud fraction (CF) changes in a warming climate. As in Fig. 4 but with the annual scale GPP-to-CF sensitivity used for the estimations derived from Supplementary Fig. 32a based on GPP and CF data from the Moderate Resolution Imaging Spectroradiometer (MODIS) onboard Terra.

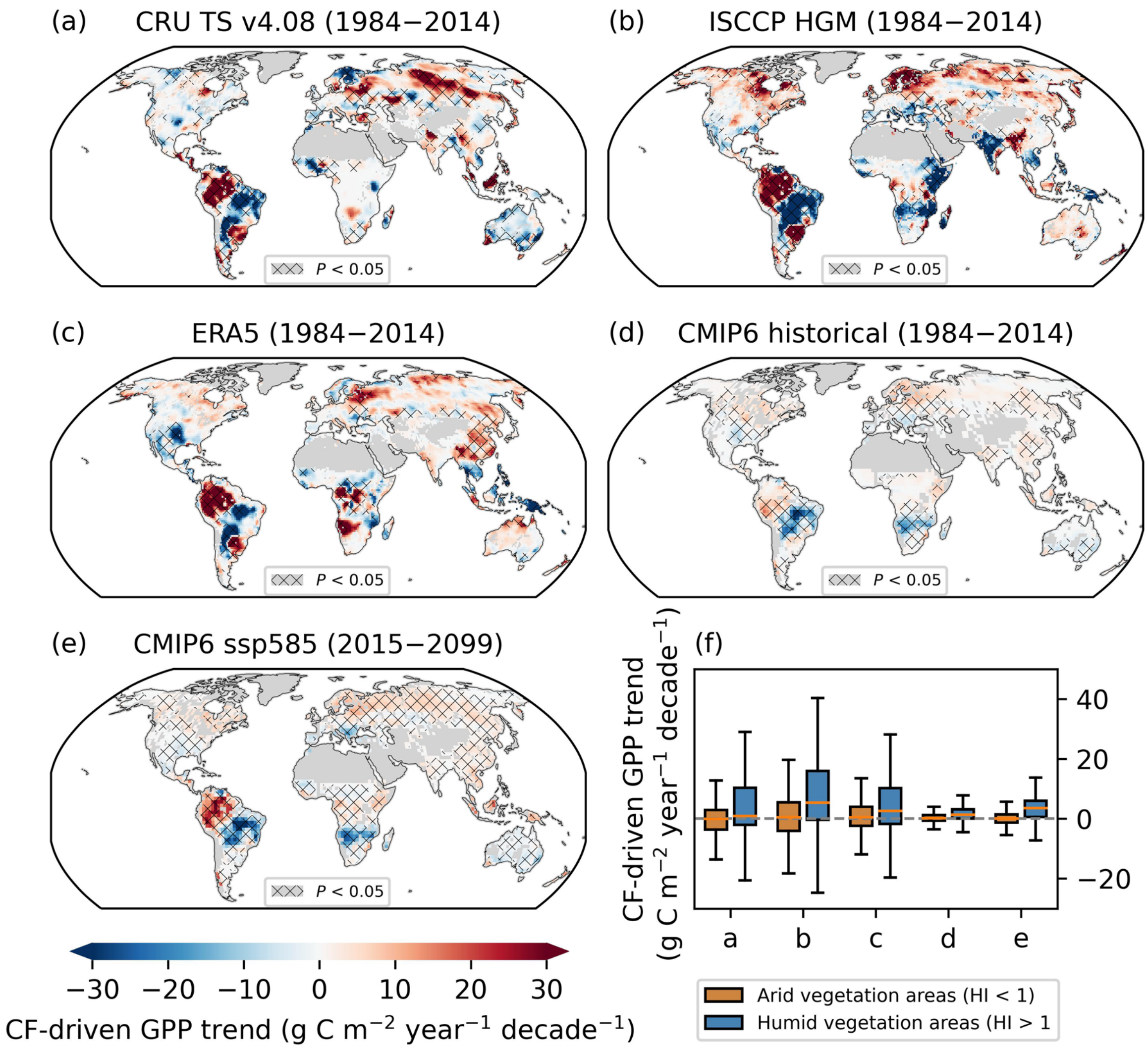

Supplementary Figure 35. Long-term trends in gross primary productivity (GPP) driven by cloud fraction (CF). As in Supplementary Fig. 17 but with the annual scale GPP-to-CF sensitivity used for the estimations derived from Supplementary Fig. 32a based on GPP and CF data from the Moderate Resolution Imaging Spectroradiometer (MODIS) onboard Terra.

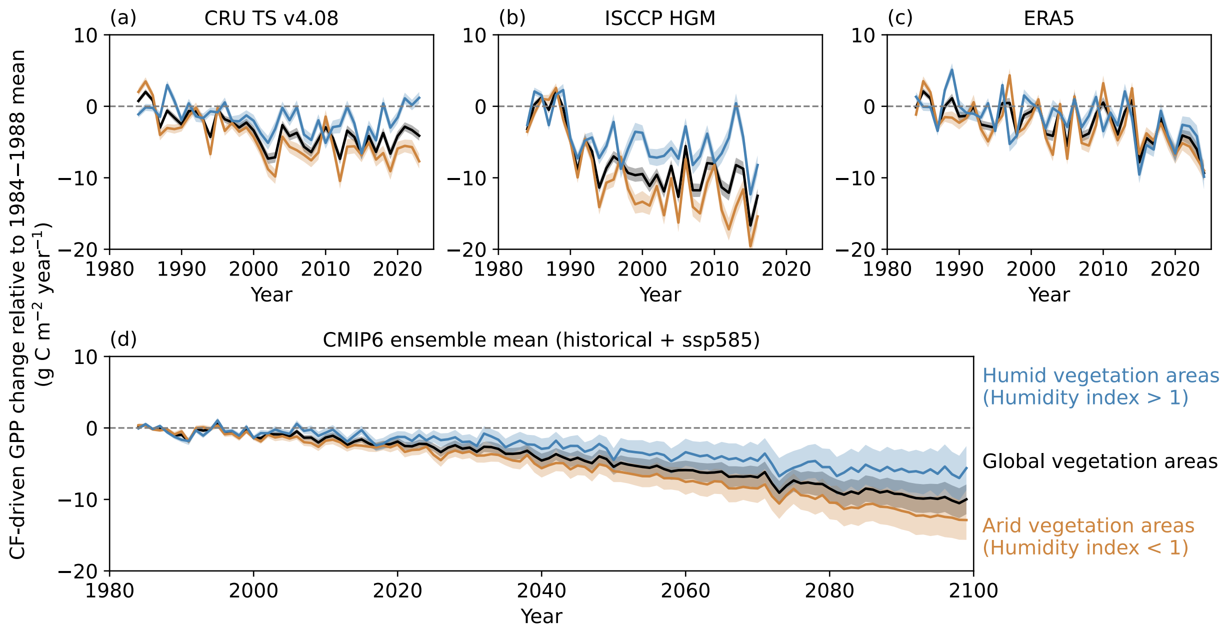

Supplementary Figure 36. Shifts in gross primary productivity (GPP) from arid to humid regions driven by cloud fraction (CF) changes in a warming climate. As in Fig. 4 but with the annual scale GPP-to-CF sensitivity used for the estimations derived from Supplementary Fig. 33a based on GPP data from FLUXCOM-X-BASE and CF data from the Moderate Resolution Imaging Spectroradiometer (MODIS) onboard Terra.

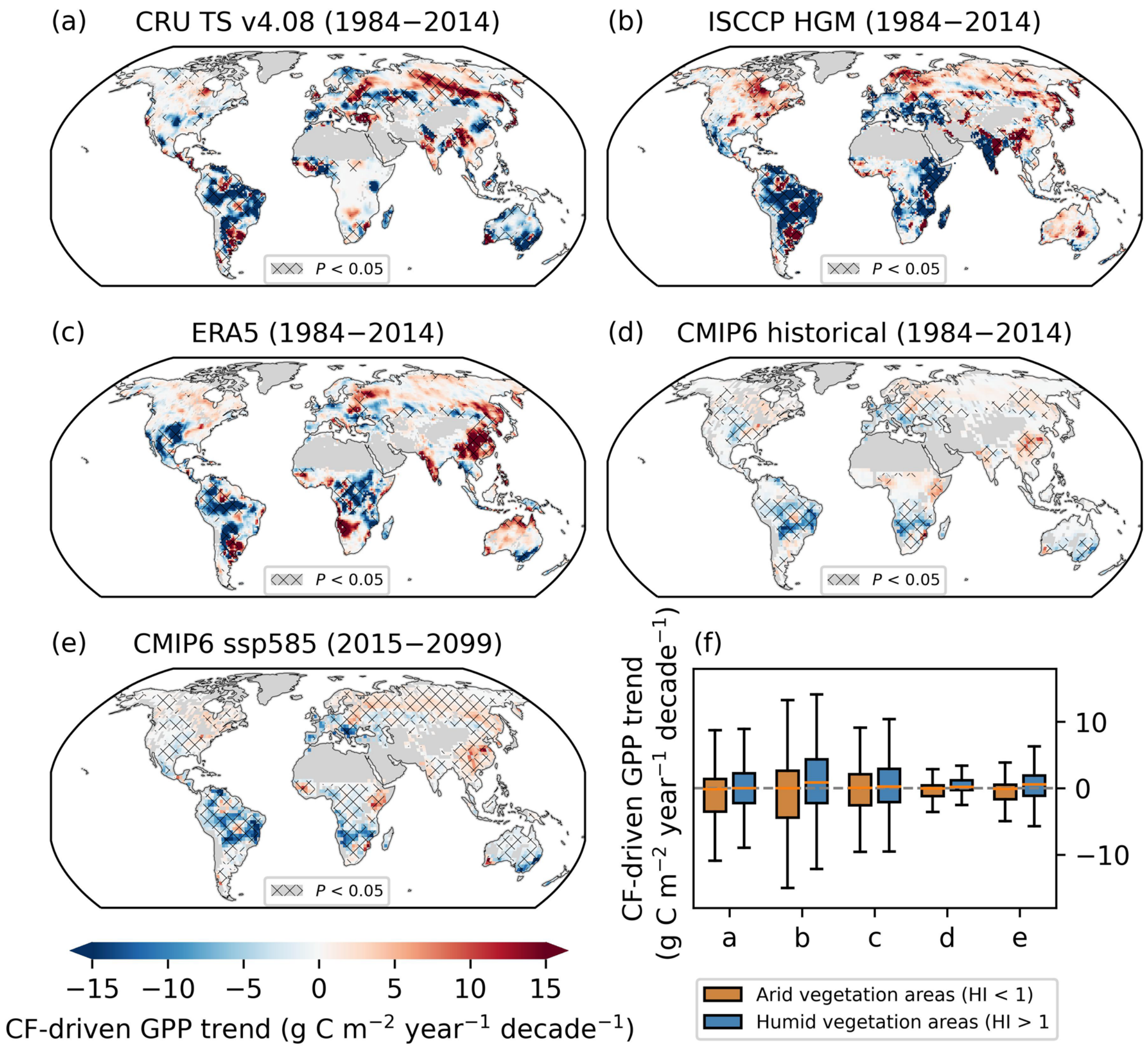

Supplementary Figure 37. Long-term trends in gross primary productivity (GPP) driven by cloud fraction (CF). As in Supplementary Fig. 17 but with the annual scale GPP-to-CF sensitivity used for the estimations derived from Supplementary Fig. 33a based on GPP data from FLUXCOM-X-BASE and CF data from the Moderate Resolution Imaging Spectroradiometer (MODIS) onboard Terra.

Supplementary Table 1. List of FLUXNET sites used in this study.

No.	Site ID	Longitude (°)	Latitude (°)	IGBP classification
1	AT-Neu	11.3175	47.1167	GRA
2	AU-ASM	133.249	–22.283	SAV
3	AU-DaP	131.3181	–14.0633	GRA
4	AU-DaS	131.3881	–14.1593	SAV
5	AU-Dry	132.3706	–15.2588	SAV
6	AU-Gin	115.7138	–31.3764	WSA
7	AU-How	131.1523	–12.4943	WSA
8	AU-Rig	145.5759	–36.6499	GRA
9	AU-Stp	133.3502	–17.1507	GRA
10	AU-Tum	148.1517	–35.6566	EBF
11	AU-Wac	145.1878	–37.4259	EBF
12	AU-Whr	145.0294	–36.6732	EBF
13	AU-Wom	144.0944	–37.4222	EBF
14	BE-Lon	4.7462	50.5516	CRO
15	BE-Vie	5.9981	50.3049	MF
16	BR-Sa3	–54.9714	–3.018	EBF
17	CA-Gro	–82.1556	48.2167	MF
18	CA-NS1	–98.4839	55.8792	ENF
19	CA-NS3	–98.3822	55.9117	ENF
20	CA-NS4	–98.3806	55.9144	ENF
21	CA-NS6	–98.9644	55.9167	OSH
22	CA-NS7	–99.9483	56.6358	OSH
23	CA-Oas	–106.198	53.6289	DBF
24	CA-Qfo	–74.3421	49.6925	ENF
25	CA-SF1	–105.818	54.485	ENF
26	CA-SF2	–105.878	54.2539	ENF
27	CA-SF3	–106.005	54.0916	OSH
28	CA-TP1	–80.5595	42.6609	ENF
29	CA-TP2	–80.4588	42.7744	ENF
30	CA-TP3	–80.3483	42.7068	ENF
31	CA-TP4	–80.3574	42.7102	ENF
32	CA-TPD	–80.5577	42.6353	DBF
33	CH-Cha	8.4104	47.2102	GRA
34	CH-Dav	9.8559	46.8153	ENF
35	CH-Fru	8.5378	47.1158	GRA
36	CH-Lae	8.3644	47.4783	MF
37	CH-Oe1	7.7319	47.2858	GRA
38	CH-Oe2	7.7337	47.2864	CRO
39	CN-Cha	128.0958	42.4025	MF
40	CN-Cng	123.5092	44.5934	GRA
41	CN-Din	112.5361	23.1733	EBF
42	CN-Ha2	101.3269	37.6086	WET
43	CN-HaM	101.18	37.37	GRA

44	CN-Qia	115.0581	26.7414	ENF
45	CZ-BK1	18.5369	49.5021	ENF
46	CZ-BK2	18.5429	49.4944	GRA
47	DE-Geb	10.9146	51.0997	CRO
48	DE-Gri	13.5126	50.95	GRA
49	DE-Hai	10.4522	51.0792	DBF
50	DE-Kli	13.5224	50.8931	CRO
51	DE-Lkb	13.3047	49.0996	ENF
52	DE-Lnf	10.3678	51.3282	DBF
53	DE-Obe	13.7213	50.7867	ENF
54	DE-Seh	6.4497	50.8706	CRO
55	DE-Tha	13.5651	50.9626	ENF
56	DK-Eng	12.1918	55.6905	GRA
57	DK-Sor	11.6446	55.4859	DBF
58	ES-LJu	–2.7521	36.9266	OSH
59	FI-Hyy	24.2948	61.8474	ENF
60	FI-Sod	26.6386	67.3624	ENF
61	FR-Gri	1.9519	48.8442	CRO
62	FR-LBr	–0.7693	44.7171	ENF
63	GF-Guy	–52.9249	5.2788	EBF
64	GH-Ank	–2.6942	5.2685	EBF
65	GL-ZaH	–20.5503	74.4733	GRA
66	IT-BCi	14.9574	40.5237	CRO
67	IT-CA1	12.0266	42.3804	DBF
68	IT-CA2	12.026	42.3772	CRO
69	IT-CA3	12.0222	42.38	DBF
70	IT-Col	13.5881	41.8494	DBF
71	IT-Cpz	12.3761	41.7052	EBF
72	IT-Lav	11.2813	45.9562	ENF
73	IT-MBo	11.0458	46.0147	GRA
74	IT-Noe	8.1517	40.6062	CSH
75	IT-Ren	11.4337	46.5869	ENF
76	IT-Ro1	11.93	42.4081	DBF
77	IT-Ro2	11.9209	42.3903	DBF
78	IT-SRo	10.2844	43.7279	ENF
79	IT-Tor	7.5781	45.8444	GRA
80	JP-SMF	137.0788	35.2617	MF
81	MY-PSO	102.3062	2.973	EBF
82	NL-Loo	5.7436	52.1666	ENF
83	RU-Fyo	32.9221	56.4615	ENF
84	SD-Dem	30.4783	13.2829	SAV
85	SN-Dhr	–15.4322	15.4028	SAV
86	US-AR1	–99.42	36.4267	GRA
87	US-AR2	–99.5975	36.6358	GRA
88	US-ARM	–97.4888	36.6058	CRO
89	US-Blo	–120.633	38.8953	ENF

90	US-CRT	–83.3471	41.6285	CRO
91	US-GLE	–106.24	41.3665	ENF
92	US-Goo	–89.8735	34.2547	GRA
93	US-IB2	–88.241	41.8406	GRA
94	US-KS2	–80.6715	28.6086	CSH
95	US-Me2	–121.559	44.4526	ENF
96	US-Me3	–121.608	44.3154	ENF
97	US-Me5	–121.567	44.4372	ENF
98	US-Me6	–121.608	44.3233	ENF
99	US-MMS	–86.4131	39.3232	DBF
100	US-NR1	–105.546	40.0329	ENF
101	US-Oho	–83.8438	41.5545	DBF
102	US-PFa	–90.2723	45.9459	MF
103	US-SRC	–110.84	31.9083	OSH
104	US-SRG	–110.828	31.7894	GRA
105	US-SRM	–110.866	31.8214	WSA
106	US-Syv	–89.3477	46.242	MF
107	US-Ton	–120.966	38.4309	WSA
108	US-UMB	–84.7138	45.5598	DBF
109	US-UMd	–84.6975	45.5625	DBF
110	US-Var	–120.951	38.4133	GRA
111	US-WCr	–90.0799	45.8059	DBF
112	US-Whs	–110.052	31.7438	OSH
113	US-Wkg	–109.942	31.7365	GRA
114	ZM-Mon	23.2525	–15.4391	DBF

Supplementary Table 2. List of the 20 dynamic global vegetation models included in the TRENDYv12 project used in this study. The S2 simulations of monthly gross primary productivity (GPP) are employed. For ISAM, we use data from TRENDYv11, as the file submitted to TRENDYv12 was corrupted at the time of our download.

No.	Model name	Grid	Reference
1	CABLE-POP	360 × 180	Haverd, et al. [4]
2	CLASSIC	360 × 180	Melton, et al. [5]
3	CLM5.0	288 × 192	Lawrence, et al. [6]
4	DLEM	720 × 360	Tian, et al. [7]
5	E3SM	288 × 192	Ricciuto, et al. [8]
6	EDv3	720 × 360	Ma, et al. [9]
7	IBIS	720 × 360	Foley, et al. [10]
8	ISAM	720 × 360	Jain, et al. [11]
9	ISBA-CTRIP	360 × 150	Delire, et al. [12]
10	JSBAC	192 × 96	Thum, et al. [13]
11	JULES	192 × 144	McNeall, et al. [14]
12	LPJ-GUESS	720 × 360	Gregor, et al. [15]
13	LPJmL	720 × 360	Bondeau, et al. [16]
14	LPJwsl	720 × 360	Calle and Poulter [17]
15	LPX-Bern	720 × 360	Sun and Joos [18]
16	OCN	360 × 180	Zaehle and Friend [19]
17	ORCHIDEE	720 × 360	Krinner, et al. [20]
18	SDGVM	360 × 180	Woodward and Lomas [21]
19	VISIT	720 × 360	Ito and Inatomi [22]
20	YIBs	360 × 181	Yue and Unger [23]

Supplementary Table 3. List of the sixth phase Coupled Model Intercomparison Project (CMIP6) 39 models used in this study. The monthly cloud fraction (CF) data are used during the historical period (1984–2014) and projected period (2015–2099). Scenario "ssp585" is employed in the projected period. All the simulations have the variant label "r1i1p1f1".

No.	Model name	Grid	Reference
1	ACCESS-CM2	192 × 144	Bi, et al. [24]
2	ACCESS-ESM1-5	192 × 144	Ziehn, et al. [25]
3	AWI-CM-1-1-MR	384 × 192	Semmler, et al. [26]
4	AWI-ESM-1-REcoM	192 × 96	Ackermann, et al. [27]
5	BCC-CSM2-MR	320 × 160	Wu, et al. [28]
6	CAMS-CSM1-0	320 × 160	Rong, et al. [29]
7	CAS-ESM2-0	256 × 128	Zhang, et al. [30]
8	CESM2-WACCM	288 × 192	Danabasoglu, et al. [31]
9	CIESM	288 × 192	Lin, et al. [32]
10	CMCC-CM2-SR5	288 × 192	Cherchi, et al. [33]
11	CMCC-ESM2	288 × 192	Lovato, et al. [34]
12	CanESM5	128 × 64	Swart, et al. [35]
13	CanESM5-1	128 × 64	Swart, et al. [35]
14	E3SM-1-0	360 × 180	Golaz, et al. [36]
15	E3SM-1-1	360 × 180	Golaz, et al. [36]
16	E3SM-1-1-ECA	360 × 180	Golaz, et al. [36]
17	EC-Earth3	512 × 256	Döscher, et al. [37]
18	EC-Earth3-CC	512 × 256	Döscher, et al. [37]
19	EC-Earth3-Veg	512 × 256	Döscher, et al. [37]
20	EC-Earth3-Veg-LR	320 × 160	Döscher, et al. [37]
21	FGOALS-f3-L	288 × 180	He, et al. [38]
22	FGOALS-g3	180 × 80	Li, et al. [39]
23	FIO-ESM-2-0	288 × 192	Bao, et al. [40]
24	GFDL-CM4	288 × 180	Held, et al. [41]
25	GFDL-ESM4	288 × 180	Dunne, et al. [42]
26	IITM-ESM	192 × 94	Krishnan, et al. [43]
27	INM-CM4-8	180 × 120	Volodin, et al. [44]
28	INM-CM5-0	180 × 120	Volodin, et al. [45]
29	IPSL-CM6A-LR	144 × 143	Boucher, et al. [46]
30	KACE-1-0-G	192 × 144	Lee, et al. [47]
31	KIOST-ESM	192 × 96	Pak, et al. [48]
32	MIROC6	256 × 128	Tatebe, et al. [49]
33	MPI-ESM1-2-HR	384 × 192	Müller, et al. [50]
34	MPI-ESM1-2-LR	192 × 96	Mauritsen, et al. [51]
35	MRI-ESM2-0	320 × 160	Yukimoto, et al. [52]
36	NESM3	192 × 96	Cao, et al. [53]
37	NorESM2-LM	144 × 96	Seland, et al. [54]
38	NorESM2-MM	288 × 192	Seland, et al. [54]

39	TaiESM1	288 × 192	Wang, et al. [55]

Supplementary references

1 Nelson, J. A. *et al.* X-BASE: the first terrestrial carbon and water flux products from an extended data-driven scaling framework, FLUXCOM-X. *Biogeosciences* **21**, 5079-5115, doi:10.5194/bg-21-5079-2024 (2024).

2 Teubner, I. E. *et al.* Assessing the relationship between microwave vegetation optical depth and gross primary production. *International Journal of Applied Earth Observation and Geoinformation* **65**, 79-91, doi:10.1016/j.jag.2017.10.006 (2018).

3 Moesinger, L. *et al.* The global long-term microwave Vegetation Optical Depth Climate Archive (VODCA). *Earth System Science Data* **12**, 177-196, doi:10.5194/essd-12-177-2020 (2020).

4 Haverd, V. *et al.* A new version of the CABLE land surface model (Subversion revision r4601) incorporating land use and land cover change, woody vegetation demography, and a novel optimisation-based approach to plant coordination of photosynthesis. *Geoscientific Model Development* **11**, 2995-3026, doi:10.5194/gmd-11-2995-2018 (2018).

5 Melton, J. R. *et al.* CLASSIC v1.0: the open-source community successor to the Canadian Land Surface Scheme (CLASS) and the Canadian Terrestrial Ecosystem Model (CTEM) – Part 1: Model framework and site-level performance. *Geoscientific Model Development* **13**, 2825-2850, doi:10.5194/gmd-13-2825-2020 (2020).

6 Lawrence, D. M. *et al.* The Community Land Model Version 5: Description of new features, benchmarking, and impact of forcing uncertainty. *Journal of Advances in Modeling Earth Systems* **11**, 4245-4287, doi:10.1029/2018MS001583 (2019).

7 Tian, H. *et al.* North American terrestrial CO_2 uptake largely offset by CH_4 and N_2O emissions: toward a full accounting of the greenhouse gas budget. *Climatic Change* **129**, 413-426, doi:10.1007/s10584-014-1072-9 (2015).

8 Ricciuto, D., Sargsyan, K. & Thornton, P. The impact of parametric uncertainties on biogeochemistry in the E3SM land model. *Journal of Advances in Modeling Earth Systems* **10**, 297-319, doi:10.1002/2017MS000962 (2018).

9 Ma, L. *et al.* Global evaluation of the Ecosystem Demography model (ED v3.0). *Geoscientific Model Development* **15**, 1971-1994, doi:10.5194/gmd-15-1971-2022 (2022).

10 Foley, J. A., Kucharik, C. J. & Polzin, D. Integrated Biosphere Simulator Model (IBIS), Version 2.5. *ORNL Distributed Active Archive Center*, doi:10.3334/ORNLDAAC/808 (2005).

11 Jain, A. K., Kheshgi, H. S. & Wuebbles, D. J. A globally aggregated reconstruction of cycles of carbon and its isotopes. *Tellus B* **48**, 583-600, doi:10.1034/j.1600-0889.1996.t01-1-00012.x (1996).

12 Delire, C. *et al.* The global land carbon cycle simulated with ISBA-CTRIP: Improvements over the last decade. *Journal of Advances in Modeling Earth Systems* **12**, e2019MS001886, doi:10.1029/2019MS001886 (2020).

13 Thum, T. *et al.* Soil carbon model alternatives for ECHAM5/JSBACH climate model: Evaluation and impacts on global carbon cycle estimates. *Journal of Geophysical Research: Biogeosciences* **116**, doi:10.1029/2010JG001612 (2011).

14 McNeall, D., Robertson, E. & Wiltshire, A. Constraining the carbon cycle in JULES-ES-1.0. *Geoscientific Model Development* **17**, 1059-1089, doi:10.5194/gmd-17-1059-2024 (2024).

15 Gregor, K. *et al.* Quantifying the impact of key factors on the carbon mitigation potential of managed temperate forests. *Carbon Balance and Management* **19**, 10, doi:10.1186/s13021-023-00247-9 (2024).

16 Bondeau, A. *et al.* Modelling the role of agriculture for the 20th century global terrestrial carbon balance. *Global Change Biology* **13**, 679-706, doi:10.1111/j.1365-2486.2006.01305.x (2007).

17 Calle, L. & Poulter, B. Ecosystem age-class dynamics and distribution in the LPJ-wsl v2.0 global ecosystem model. *Geoscientific Model Development* **14**, 2575-2601, doi:10.5194/gmd-14-2575-2021 (2021).

18 Sun, Q. & Joos, F. Modelling terrestrial vegetation dynamics and carbon-nitrogen cycles over the last glacial using LPX-Bern. *EGU General Assembly Conference Abstracts*, 10571, doi:10.5194/egusphere-egu24-10571 (2024).

19 Zaehle, S. & Friend, A. D. Carbon and nitrogen cycle dynamics in the O-CN land surface model: 1. Model description, site-scale evaluation, and sensitivity to parameter estimates. *Global Biogeochemical Cycles* **24**, doi:10.1029/2009GB003521 (2010).

20 Krinner, G. *et al.* A dynamic global vegetation model for studies of the coupled atmosphere-biosphere system. *Global Biogeochemical Cycles* **19**, doi:10.1029/2003GB002199 (2005).

21 Woodward, F. I. & Lomas, M. R. Vegetation dynamics – simulating responses to climatic change. *Biological Reviews* **79**, 643-670, doi:10.1017/S1464793103006419 (2004).

22 Ito, A. & Inatomi, M. Use of a process-based model for assessing the methane budgets of global terrestrial ecosystems and evaluation of uncertainty. *Biogeosciences* **9**, 759-773, doi:10.5194/bg-9-759-2012 (2012).

23 Yue, X. & Unger, N. The Yale Interactive terrestrial Biosphere model version 1.0: description, evaluation and implementation into NASA GISS ModelE2. *Geoscientific Model Development* **8**, 2399-2417, doi:10.5194/gmd-8-2399-2015 (2015).

24 Bi, D. *et al.* Configuration and spin-up of ACCESS-CM2, the new generation Australian Community Climate and Earth System Simulator Coupled Model. *Journal of Southern Hemisphere Earth Systems Science* **70**, 225-251, doi:10.1071/ES19040 (2020).

25 Ziehn, T. *et al.* The Australian Earth System Model: ACCESS-ESM1.5. *Journal of Southern Hemisphere Earth Systems Science* **70**, 193-214, doi:10.1071/ES19035 (2020).

26 Semmler, T. *et al.* Simulations for CMIP6 with the AWI climate model AWI-CM-1-1. *Journal of Advances in Modeling Earth Systems* **12**, e2019MS002009, doi:10.1029/2019MS002009 (2020).

27 Ackermann, L. *et al.* A comprehensive Earth system model (AWI-ESM2.1) with interactive icebergs: effects on surface and deep-ocean characteristics. *Geoscientific Model Development* **17**, 3279-3301, doi:10.5194/gmd-17-3279-2024 (2024).

28 Wu, T. *et al.* BCC-CSM2-HR: a high-resolution version of the Beijing Climate Center Climate System Model. *Geoscientific Model Development* **14**, 2977-3006, doi:10.5194/gmd-14-2977-2021 (2021).

29 Rong, X. *et al.* The CAMS climate system model and a basic evaluation of its climatology and climate variability simulation. *Journal of Meteorological Research* **32**, 839-861, doi:10.1007/s13351-018-8058-x (2018).

30 Zhang, H. *et al.* Description and climate simulation performance of CAS-ESM Version 2. *Journal of Advances in Modeling Earth Systems* **12**, e2020MS002210, doi:10.1029/2020MS002210 (2020).

31 Danabasoglu, G. *et al.* The Community Earth System Model Version 2 (CESM2). *Journal of Advances in Modeling Earth Systems* **12**, e2019MS001916, doi:10.1029/2019MS001916 (2020).

32 Lin, Y. *et al.* Community Integrated Earth System Model (CIESM): Description and evaluation. *Journal of Advances in Modeling Earth Systems* **12**, e2019MS002036, doi:10.1029/2019MS002036 (2020).

33 Cherchi, A. *et al.* Global mean climate and main patterns of variability in the CMCC-CM2 coupled model. *Journal of Advances in Modeling Earth Systems* **11**, 185-209, doi:10.1029/2018MS001369 (2019).

34 Lovato, T. *et al.* CMIP6 simulations with the CMCC Earth System Model (CMCC-ESM2). *Journal of Advances in Modeling Earth Systems* **14**, e2021MS002814, doi:10.1029/2021MS002814 (2022).

35 Swart, N. C. *et al.* The Canadian Earth System Model version 5 (CanESM5.0.3). *Geoscientific Model Development* **12**, 4823-4873, doi:10.5194/gmd-12-4823-2019 (2019).

36 Golaz, J.-C. *et al.* The DOE E3SM Coupled Model Version 1: Overview and evaluation at standard resolution. *Journal of Advances in Modeling Earth Systems* **11**, 2089-2129, doi:10.1029/2018MS001603 (2019).

37 Döscher, R. *et al.* The EC-Earth3 Earth system model for the Coupled Model Intercomparison Project 6. *Geoscientific Model Development* **15**, 2973-3020, doi:10.5194/gmd-15-2973-2022 (2022).

38 He, B. *et al.* CAS FGOALS-f3-L model datasets for CMIP6 historical atmospheric model intercomparison project simulation. *Advances in Atmospheric Sciences* **36**, 771-778, doi:10.1007/s00376-019-9027-8 (2019).

39 Li, L. *et al.* The Flexible Global Ocean-Atmosphere-Land System Model Grid-Point Version 3 (FGOALS-g3): Description and evaluation. *Journal of Advances in Modeling Earth Systems* **12**, e2019MS002012, doi:10.1029/2019MS002012 (2020).

40 Bao, Y., Song, Z. & Qiao, F. FIO-ESM Version 2.0: Model description and evaluation. *Journal of Geophysical Research: Oceans* **125**, e2019JC016036, doi:10.1029/2019JC016036 (2020).

41 Held, I. M. *et al.* Structure and performance of GFDL's CM4.0 climate model. *Journal of Advances in Modeling Earth Systems* **11**, 3691-3727, doi:10.1029/2019MS001829 (2019).

42 Dunne, J. P. *et al.* The GFDL Earth System Model Version 4.1 (GFDL-ESM 4.1): Overall coupled model description and simulation characteristics. *Journal of Advances in Modeling Earth Systems* **12**, e2019MS002015, doi:10.1029/2019MS002015 (2020).

43 Krishnan, R. *et al.* The IITM Earth System Model (IITM ESM). *arXiv preprint*, doi:arxiv.org/abs/2101.03410 (2021).

44 Volodin, E. M. *et al.* Simulation of the modern climate using the INM-CM48 climate model. *Russian Journal of Numerical Analysis and Mathematical Modelling* **33**, 367-374, doi:10.1515/rnam-2018-0032 (2018).

45 Volodin, E. M. *et al.* Simulation of the present-day climate with the climate model INMCM5. *Climate Dynamics* **49**, 3715-3734, doi:10.1007/s00382-017-3539-7 (2017).

46 Boucher, O. *et al.* Presentation and evaluation of the IPSL-CM6A-LR climate model. *Journal of Advances in Modeling Earth Systems* **12**, e2019MS002010, doi:10.1029/2019MS002010 (2020).

47 Lee, J. *et al.* Evaluation of the Korea Meteorological Administration Advanced Community Earth-System model (K-ACE). *Asia-Pacific Journal of Atmospheric Sciences* **56**, 381-395, doi:10.1007/s13143-019-00144-7 (2020).

48 Pak, G. *et al.* Korea Institute of Ocean Science and Technology Earth System Model and its simulation characteristics. *Ocean Science Journal* **56**, 18-45, doi:10.1007/s12601-021-00001-7 (2021).

49 Tatebe, H. *et al.* Description and basic evaluation of simulated mean state, internal variability, and climate sensitivity in MIROC6. *Geoscientific Model Development* **12**, 2727-2765, doi:10.5194/gmd-12-2727-2019 (2019).

50 Müller, W. A. *et al.* A Higher-resolution Version of the Max Planck Institute Earth System Model (MPI-ESM1.2-HR). *Journal of Advances in Modeling Earth Systems* **10**, 1383-1413, doi:10.1029/2017MS001217 (2018).

51 Mauritsen, T. *et al.* Developments in the MPI-M Earth System Model version 1.2 (MPI-ESM1.2) and its response to increasing CO_2. *Journal of Advances in Modeling Earth Systems* **11**, 998-1038, doi:10.1029/2018MS001400 (2019).

52 Yukimoto, S. *et al.* The Meteorological Research Institute Earth System Model Version 2.0, MRI-ESM2.0: Description and basic evaluation of the physical component. *Journal of the Meteorological Society of Japan. Ser. II* **97**, 931-965, doi:10.2151/jmsj.2019-051 (2019).

53 Cao, J. *et al.* The NUIST Earth System Model (NESM) version 3: description and preliminary evaluation. *Geoscientific Model Development* **11**, 2975-2993, doi:10.5194/gmd-11-2975-2018 (2018).

54 Seland, Ø. *et al.* Overview of the Norwegian Earth System Model (NorESM2) and key climate response of CMIP6 DECK, historical, and scenario simulations. *Geoscientific Model Development* **13**, 6165-6200, doi:10.5194/gmd-13-6165-2020 (2020).

55 Wang, Y.-C. *et al.* Performance of the Taiwan Earth System Model in simulating climate variability compared with observations and CMIP6 model simulations. *Journal of Advances in Modeling Earth Systems* **13**, e2020MS002353, doi:10.1029/2020MS002353 (2021).
